# A single intranasal dose of human parainfluenza virus type 3-vectored vaccine induces effective antibody and memory T cell response in the lungs and protects hamsters against SARS-CoV-2

**DOI:** 10.1038/s41541-022-00471-3

**Published:** 2022-04-25

**Authors:** Philipp A. Ilinykh, Sivakumar Periasamy, Kai Huang, Natalia A. Kuzmina, Palaniappan Ramanathan, Michelle N. Meyer, Chad E. Mire, Ivan V. Kuzmin, Preeti Bharaj, Jessica R. Endsley, Maria Chikina, Stuart C. Sealfon, Steven G. Widen, Mark A. Endsley, Alexander Bukreyev

**Affiliations:** 1 Department of Pathology, University of Texas Medical Branch, Galveston, TX USA; 2 Galveston National Laboratory, Galveston, TX USA; 3 Department of Microbiology and Immunology, University of Texas Medical Branch, Galveston, TX USA; 4 Department of Computational and Systems Biology, School of Medicine, University of Pittsburgh, Pittsburgh, PA USA; 5 Department of Neurology, Icahn School of Medicine at Mount Sinai, New York, NY USA; 6 Department of Biochemistry and Molecular Biology, University of Texas Medical Branch, Galveston, TX USA

**Keywords:** Live attenuated vaccines, SARS-CoV-2, Viral infection, Acute inflammation

## Abstract

Respiratory tract vaccination has an advantage of needle-free delivery and induction of mucosal immune response in the portal of SARS-CoV-2 entry. We utilized human parainfluenza virus type 3 vector to generate constructs expressing the full spike (S) protein of SARS-CoV-2, its S1 subunit, or the receptor-binding domain, and tested them in hamsters as single-dose intranasal vaccines. The construct bearing full-length S induced high titers of neutralizing antibodies specific to S protein domains critical to the protein functions. Robust memory T cell responses in the lungs were also induced, which represent an additional barrier to infection and should be less sensitive than the antibody responses to mutations present in SARS-CoV-2 variants. Following SARS-CoV-2 challenge, animals were protected from the disease and detectable viral replication. Vaccination prevented induction of gene pathways associated with inflammation. These results indicate advantages of respiratory vaccination against COVID-19 and inform the design of mucosal SARS-CoV-2 vaccines.

## Introduction

In December 2019, an outbreak of a severe respiratory disease was first reported in the city of Wuhan, Hubei, China. The causative agent of this outbreak was identified as a novel coronavirus named severe acute respiratory syndrome coronavirus-2 (SARS-CoV-2), causing COVID-19^1^. The World Health Organization declared the outbreak a Public Health Emergency of International Concern on January 30, 2020, and a pandemic on March 11, 2020. It spread rapidly around the world, causing more than 352 million cases and 5.6 million deaths as of January 25, 2022 (https://covid19.who.int). Since the last quarter of 2020, variant viruses have emerged in many parts of the world as a result of the high burden of infection and the adaptation of SARS-CoV-2 to human cells under immune pressure^2,3^. While approved SARS-CoV-2 vaccines are being rolled out, many developing countries are still waiting for access to these vaccines. Even with the deployment of safe and effective vaccines, alternative vaccine platforms are needed to address the pandemic^4^. Furthermore, children and infants, who were considered less susceptible at the beginning of the pandemic, are now representing an important population which requires vaccination^5^.

Neutralizing antibody titers are likely to be an essential correlate of protection against SARS-CoV-2^6^. This was further confirmed in the clinical trials of several vaccine candidates^7^. The envelope spike (S) glycoprotein of SARS-CoV-2, which enables binding and entry to the host cell, is comprised of two subunits, S1 and S2. The S1 subunit contains the receptor-binding domain (RBD), which is responsible for recognition of the carboxypeptidase angiotensin-converting enzyme 2 (ACE2) receptor on host cells. Being the sole viral antigen that elicits the neutralizing immune response, the S protein serves as a main target for therapeutic antibodies and vaccine design efforts. Its RBD contains numerous conformational B cell epitopes^8^. RBD-specific antibodies prevent virus attachment to the host cell and were shown to make up most of the virus-neutralizing response during infection^9–12^.

The initial site of SARS-CoV-2 infection is the sinonasal epithelium^13^. The pathogenesis of the early stages of COVID-19 is associated with the penetration of the upper respiratory tract by SARS-CoV-2 and the subsequent development of viral infection in tissues of the upper and lower respiratory tracts. The level of lung damage largely determines the severity and outcome of the disease. Therefore, the local immune responses, i.e., the S-specific antibodies on the airway mucosa and T cell immunity, play an important role in prevention of the disease by blocking SARS-CoV-2 infection upon its entry to the respiratory tract. A desirable feature of any COVID-19 vaccine is to stop viral replication in the upper respiratory tract before progression into the lungs. This feature would also strengthen prevention of the interpersonal transmission. Available vaccines against SARS-CoV-2 include those based on mRNA^14,15^, viral vectors expressing the S protein^16–19^, inactivated whole virus^20,21^, protein subunit^22^ or DNA platforms^23,24^, among others. Notably, currently approved vaccines are administered by intramuscular (IM) injection, resulting in robust systemic yet uncertain mucosal immunity. In contrast, intranasal (IN) administration has a great potential to elicit both systemic and local responses with the ease of vaccination, including the production of IgA and stimulation of T and B cells in the nasopharynx-associated lymphoid tissue^25^, that can effectively and immediately eliminate viruses entering the upper respiratory tract. Additionally, IN vaccines provide strategies for improved booster vaccinations after the prime with any of the approved vaccines, since it might also be more effective if sequential vaccinations employ different routes of administration.

Human parainfluenza virus type 3 (HPIV3) (family *Paramyxoviridae*) is an enveloped virus with a single-stranded negative-sense RNA genome. It is a common pediatric virus which infects the respiratory tract causing a mild respiratory disease and does not spread to other tissues. These features make HPIV3-based vaccines suitable for IN administration, but also ensure greater safety^26^. Therefore, HPIV3 is well-suited as a vector for pathogens that use the respiratory tract as a portal of entry, such as SARS-CoV-2. In this study, we designed a panel of constructs based on an existing JS strain of HPIV3, which is naturally attenuated in humans^27^. We generated constructs expressing the full S protein of SARS-CoV-2, its S1 subunit, or RBD expressed from an added transcriptional unit. All constructs were successfully recovered and shown to produce the expected proteins in infected cells. The efficiency of vaccine constructs was tested in a hamster animal model as a single IN dose. The full S-containing vaccine, HPIV3/full S, was able to induce strong neutralizing antibody titers and memory T cell responses resulting in barely detectable SARS-CoV-2 replication in the lungs and alleviated tissue pathologic changes and body weight loss upon challenge. Importantly, the cytotoxic CD8^+^ T cell response elicited by the vaccine in the lungs represents an additional barrier against the virus. Furthermore, this barrier is expected to be less sensitive to mutations present in SARS-CoV-2 variants than antibody responses.

## Results

### Introduction of SARS-CoV-2 S gene or its fragments into HPIV3 full-length clone results in recovery of replication-competent vaccine constructs

**Fig. 1:**
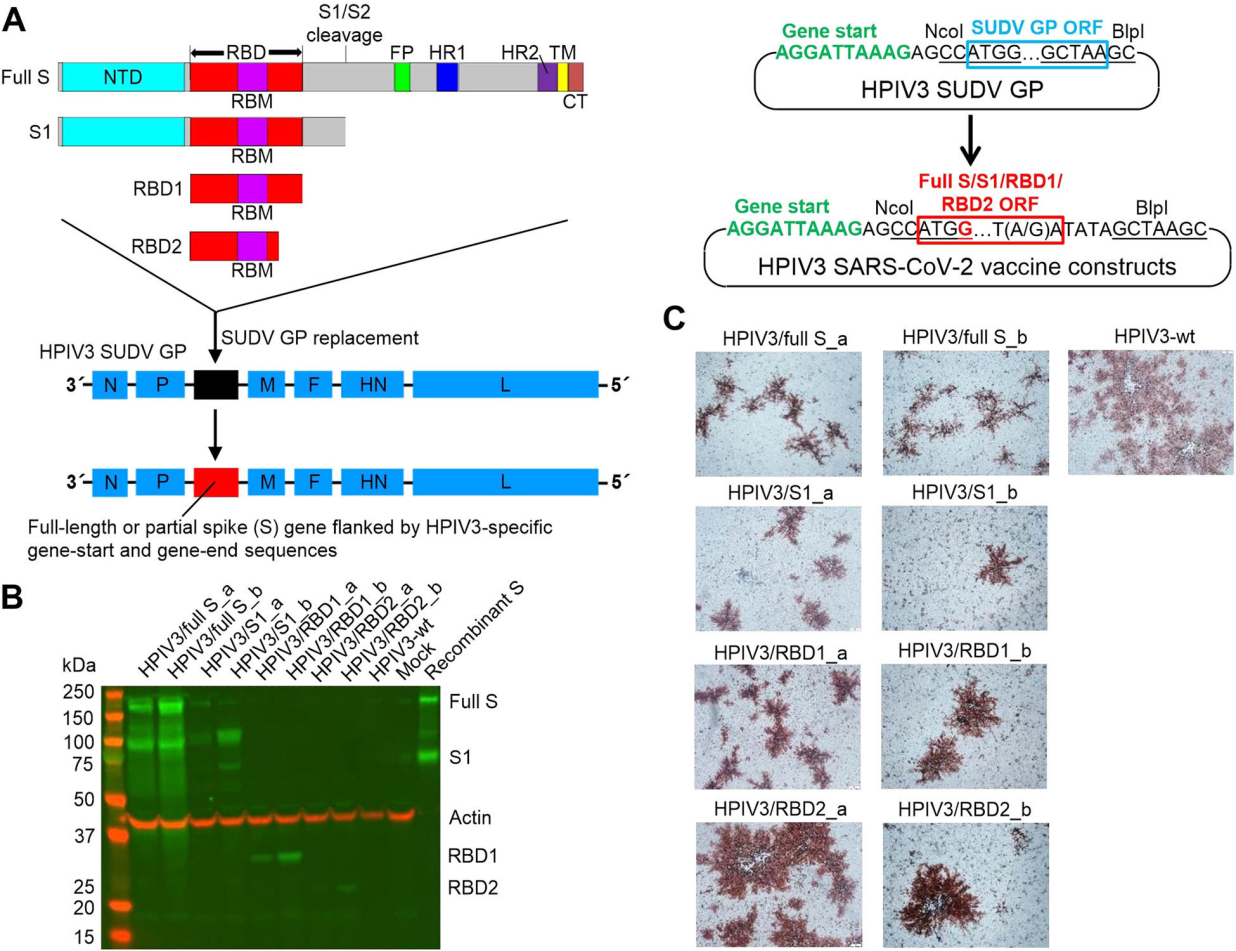
Recovery and characterization of HPIV3-vectored vaccines expressing SARS-CoV-2 S proteins. **A** Generation of vaccine constructs. The schematic domain organization of the S inserts (full S, S1, RBD1, RBD2) is shown. NTD N-terminal domain, RBD receptor-binding domain, RBM receptor-binding motif, FP fusion peptide, HR1 heptad repeat 1, HR2 heptad repeat 2, TM transmembrane domain, CT cytoplasmic tail. HPIV3 proteins: N nucleocapsid protein, P phosphoprotein, M matrix protein, F fusion protein, HN hemagglutinin-neuraminidase, L RNA polymerase. **B** LLC-MK2 cell monolayers were infected for 48 h, lysed, separated on a Western blot and stained for S proteins. Positive control: the recombinant S protein; loading control: actin. **C** Viral plaques immunostained with rabbit polyclonal antibodies against HPIV3 in LLC-MK2 cells (8 dpi).

To generate candidate vectored vaccines, we utilized HPIV3-based platform. We generated SARS-CoV-2 S transcriptional cassette by adding HPIV3-specific gene-start and gene-end transcriptional signals upstream and downstream the open-reading frame and incorporated it in the P-M intergenic sequence (Fig. 1A). We also generated the constructs expressing S1 and RBD. Since the amino acid positions determining RBD location do vary across published data, we generated constructs expressing the “long” (RBD1, 319–591 aa) and “short” (RBD2, 319–529 aa) versions of RBD^28,29^. Two versions of the S gene cDNA were used: codon-optimized and not optimized for eucaryotic translation, resulting in set “a” and set “b” constructs, respectively. Totally, eight constructs were obtained (see the section “Methods”): HPIV3/full S_a, HPIV3/full S_b, HPIV3/S1_a, HPIV3/S1_b, HPIV3/RBD1_a, HPIV3/RBD1_b, HPIV3/RBD2_a and HPIV3/RBD2_b. All of them were successfully recovered upon transfection of BSR-T7 cells. Expression of the expected S proteins in virus-infected cells was confirmed by western blot (Fig. 1B). In LLC-MK2 cells infected with HPIV3/full S constructs (the first 2 lanes), both full-length S and its cleaved product, S1, were detected, indicating the expected cleavage of the S protein. All constructs were shown to form viral plaques immunostained with HPIV3-specific antibodies in LLC-MK2 cell monolayers (Fig. 1C). As expected, the plaque size increased from full S to S1, RBD1, and RBD2 according to the reduction of length of the inserted S gene fragment.

### The full S vaccine induces a robust antibody response

**Fig. 2:**
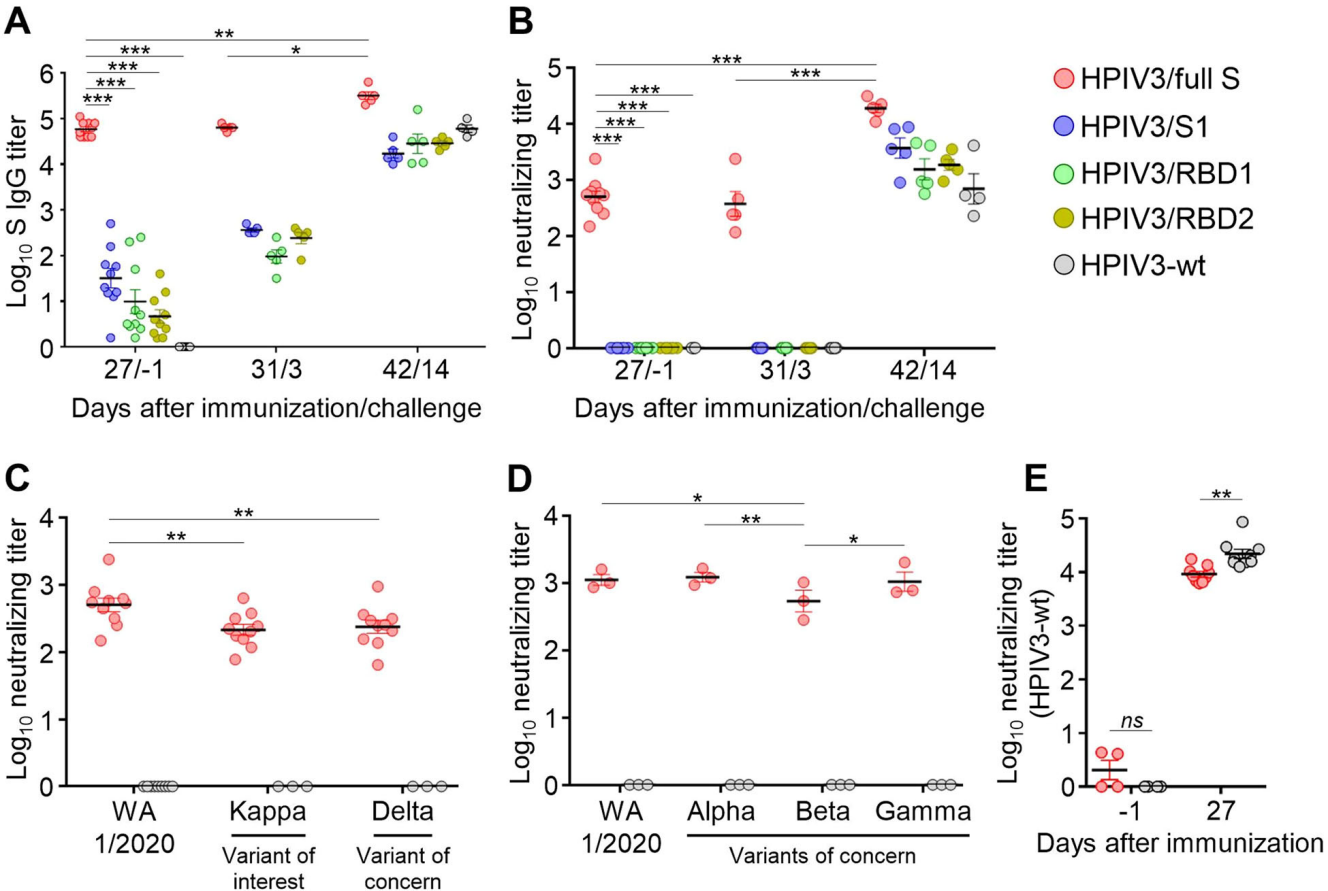
Full S vaccine elicits antibodies neutralizing SARS-CoV-2 and its genetic variants. **A** Total serum titers of SARS-CoV-2 S-specific IgG measured by ELISA. OD values from pre-bleed serum samples were subtracted from that of the test controls, and titers were determined using a four-parameter logistic curve fit. **B** Serum neutralization of mNeonGreen-expressing SARS-CoV-2 (WA1/2020) measured by high-throughput screening (HTS). **C** Serum neutralization of mNeonGreen-expressing SARS-CoV-2 (Kappa and Delta variants) on day 27 post-immunization, HTS format. Neutralization data for WA1/2020 are taken from panel B for comparison. **D** Serum neutralization of SARS-CoV-2 biological isolates on day 27 post-immunization measured by a standard plaque reduction neutralization assay. **E** Serum neutralization of HPIV3-wt (plaque reduction assay). Data represent mean ± SEM based on *n* = 3–10. *p < 0.05; **p < 0.01; ***p < 0.0001 (two-way ANOVA with multiple comparisons, Fisher’s LSD test). Ns not significant.

Since the set “b” constructs showed the uniformly higher expression of S gene fragments than the set “a” counterparts (Fig. 1B), the “b” set of constructs was further selected for immunogenicity and protection studies in the golden Syrian hamster model of SARS-CoV-2 infection (referred to without “b” index thereafter)^30^. Groups of 6–7-week-old hamsters (*n* = 10 per group) were inoculated by the IN route with single doses of the constructs expressing full S, S1, RBD1, RBD2, or wild-type (wt) HPIV3 (empty vector as mock-vaccine control) at 10^6^ plaque-forming units (PFU) per animal in a total volume of 100 µl (~50 μl into each nostril). The full S vaccine induced higher S-specific-binding antibody titers than all other vaccines (Fig. 2A). The serum neutralization assay performed with mNeonGreen SARS-CoV-2 (strain WA1/2020)^31^ revealed that only HPIV3/full S construct was able to induce neutralizing antibodies (Fig. 2B). These data show that a single IN dose of HPIV3-vectored full S vaccine induces a robust serum antibody response.

### The full S vaccine induces a robust antibody response against SARS-CoV-2 variants of concern

To evaluate the breadth of neutralization of vaccine-induced antibodies, pre-challenge samples from HPIV3/full S group were tested against Kappa and Delta SARS-CoV-2 variants (Fig. 2C). Kappa variant is classified as the variant of interest by the US CDC, whereas Delta variant is classified as the variant of concern (VOC) due to its high infectivity^32^. Samples from three animals in the HPIV3-wt group were picked randomly to serve as negative controls. Both Kappa and Delta variants were neutralized by the immune sera, although at a lower extent compared to an original WA1/2020 strain. Next, we selected three samples with the highest WA1/2020-neutralizing titers and tested them against the biological SARS-CoV-2 isolates in a standard plaque reduction neutralization assay: WA1/2020, and three VOCs, Alpha, Beta and Gamma (Fig. 2D). The level of Alpha and Gamma variants’ neutralization was comparable to that of WA1/2020, whereas Beta variant appeared to be less sensitive to serum antibodies. Finally, we tested an induction of vector-specific antibodies in response to the most immunogenic vaccine construct, HPIV3/full S, compared to HPIV3-wt used for inoculation of control animals (Fig. 2E). HPIV3-neutralizing antibody titer in the HPIV3/full S group was significantly lower compared to the HPIV3-wt group.

To summarize, we show that a single IN dose of HPIV3-vectored full S vaccine induces a robust and broad humoral immune response, which is capable of neutralizing not only the homologous virus strain, but also SARS-CoV-2 VOCs.

### The vaccine-induced antibody response targets multiple linear epitopes across the S protein

**Fig. 3:**
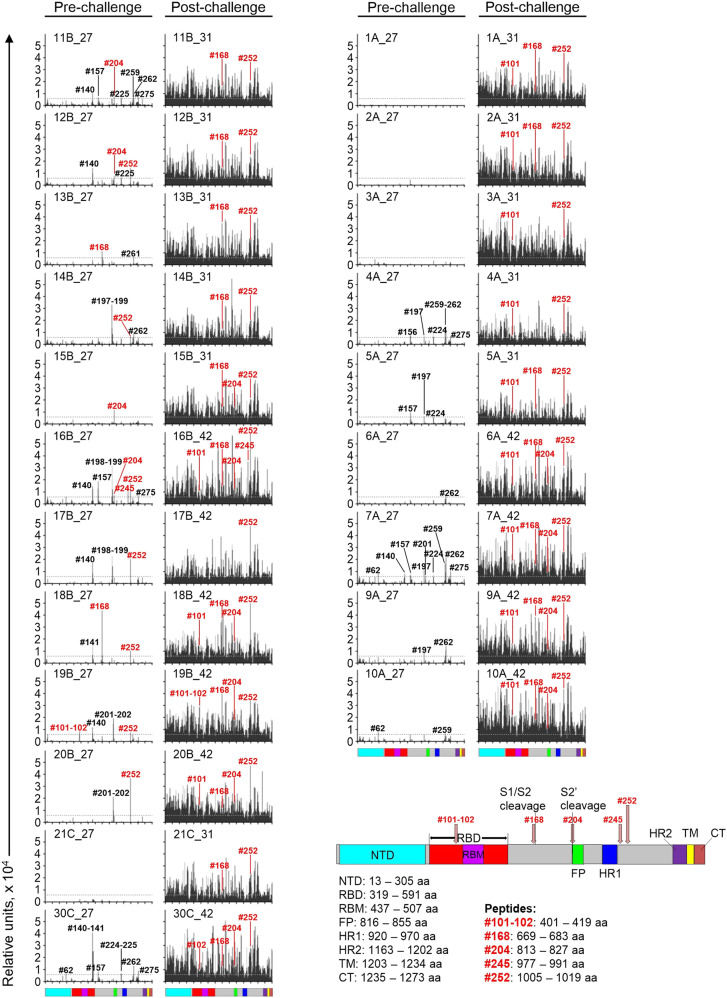
Vaccine-induced antibodies target a variety of epitopes in the S protein. The S protein linear epitopes targeted by hamster immune sera, were elucidated using peptide microarrays of immobilized 15-mer oligopeptides covering the entire SARS-CoV-2 S sequence with 4 amino acid overlaps. Data for individual animals from HPIV3-wt (1A–10A), HPIV3/full S (11B–20B) and HPIV3/S1 groups (21C, 30C) are presented. Indices show days after immunization (27, 31, 42). Each column represents MFI of a 15-residue peptide matching to the sequence of SARS-CoV-2 S. Shown are mean values of triplicate peptides (for HPIV3/full S group) or single peptide values (for other groups) after subtraction of the corresponding pre-immunization sera signals for each individual animal. Dotted lines indicate the arbitrary background level. Identification numbers are shown above the peaks for peptides exceeding background binding. Numbers in black indicate peaks that are present in HPIV3-wt control group serum samples, and numbers in red indicate peaks that are not present in the control group on day 27 post-immunization (i.e., specific for vaccine-raised antibodies). The S protein map (adapted from^33^) depicts key domains and epitopes for vaccine-induced antibodies, corresponding to the coordinates of specific peptides. The definitions for S protein domains are given in Fig. 1.

The S protein linear epitopes targeted by hamster immune sera were elucidated using peptide microarrays of immobilized 15-mer oligopeptides covering the entire SARS-CoV-2 S sequence with 4 amino acid overlaps^33,34^ (Fig. 3, Supplementary Figs. 1 and 2; Supplementary Table 1). Pre- and post-challenge samples from all animals in the HPIV3/full S and HPIV3-wt groups were analyzed. In addition, serum samples from two animals in HPIV3/S1 group with relatively high S IgG titers were also included (Fig. 2A). The slides with immobilized 15-mer oligopeptides overlapping the entire SARS-CoV-2 S sequence with 4 amino acid overlaps were incubated with serum samples, followed by incubation with secondary antibody conjugated with Cy5 fluorophore, and the mean fluorescent intensity (MFI) values for each individual peptide were recorded. The following S protein regions were identified as potential epitopes for vaccine-raised systemic IgG antibodies: 405–415 aa (the overlapping part of peptides #101 and #102; RBD, outside of the receptor-binding motif), 669–683 aa (peptide #168; in a close proximity to the S1/S2 cleavage site), 813–827 aa (peptide #204; overlapping S2′ cleavage site and N-terminus of the fusion peptide), 977–991 and 1005–1019 aa (peptides #245 and #252; adjacent to HR1). Antibodies to these S protein regions were further boosted by SARS-CoV-2 infection. Noteworthy, two samples with the highest pre-challenge neutralizing titers against the WA1/2020 strain demonstrated the presence of antibodies binding S2′ cleavage site and the fusion peptide. Seven out of 10 serum samples from HPIV3/full S group contained antibodies with binding sites located next to HR1 (Supplementary Table 1). Another potential epitope was identified in vicinity to the S1/S2 cleavage site. None of the epitopes contain any N-linked glycosylation sites^35^ or mutations identified in viral stocks of Gamma and Delta variants used for in vitro experiments (Supplementary Fig. 1). These data suggest that HPIV3-based SARS-CoV-2 vaccine induces antibodies spanning multiple linear epitopes in the S protein.

### The full S vaccine induces robust Th1 and CD8^+^ T cell responses in lungs and spleen

Separate groups of hamsters were intranasally inoculated with HPIV3/full S, HPIV3/S1 or HPIV3/RBD1 constructs, or HPIV3-wt. After 28 days, vaccinated animals were euthanized, lungs and spleen were collected, and immune cells were isolated. Multi-parameter flow cytometry analysis demonstrated that the frequencies of CD4^+^ and CD8^+^ T cells in the lungs remained unchanged in vaccinated hamsters when compared to the control hamsters vaccinated with empty vector (Supplementary Fig. 3A). However, the absolute numbers of these cells were higher in hamsters vaccinated with HPIV3/full S construct compared to other groups. In contrast to the lungs, no significant differences between HPIV3/full S and control group were observed in percentages and total T cell numbers in spleen. Similarly, the B cell numbers were higher in lungs of vaccinated animals compared to the vector-only control (Supplementary Fig. 3B). However, B cell numbers in spleen did not differ between groups.

**Fig. 4:**
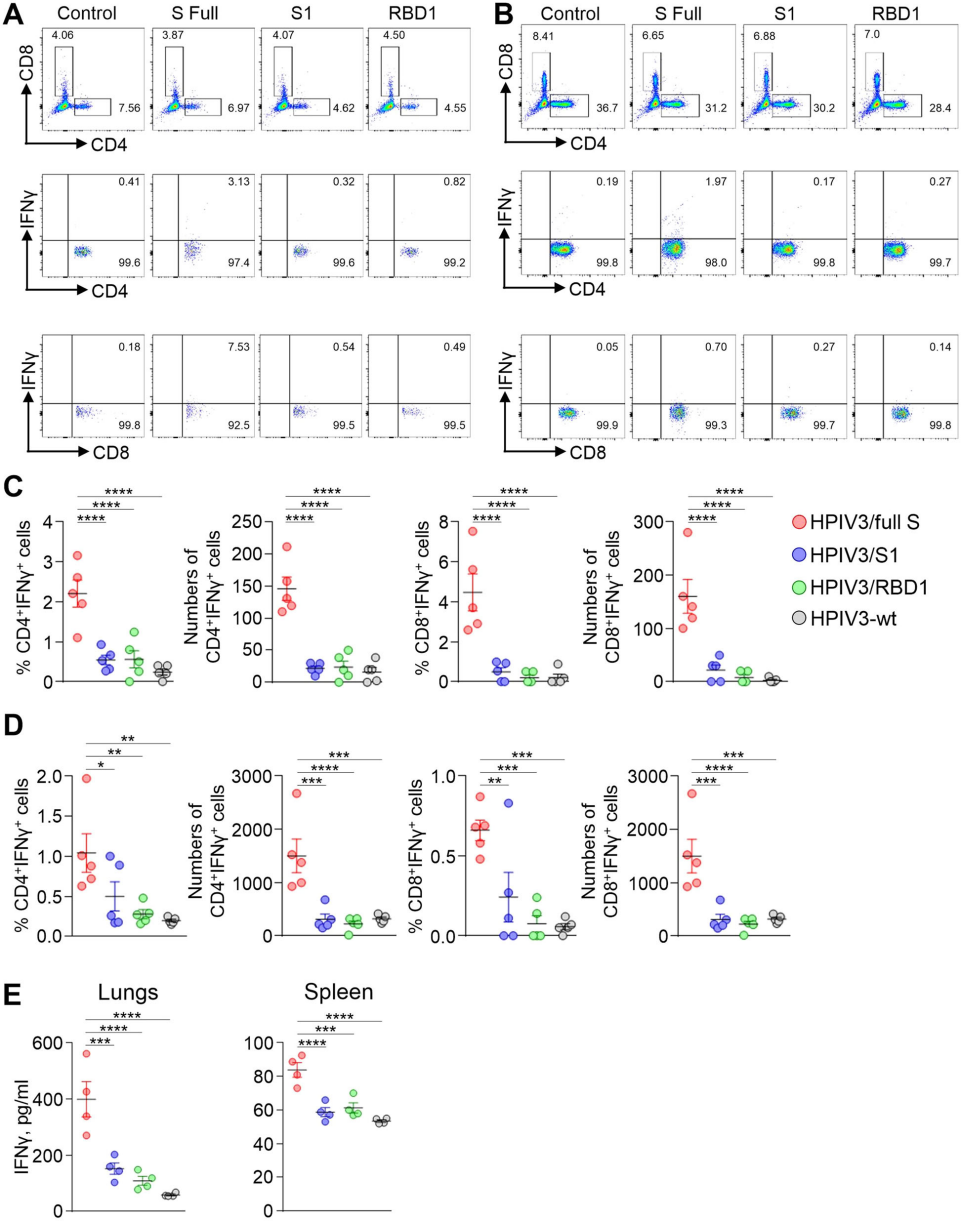
Cell-mediated response to vaccination in lungs and spleen. **A**–**D** Cells isolated from tissue samples of uninfected animals at 28 days post vaccination were stimulated with SARS-CoV-2 S-peptide pool and stained for CD4, CD8 and IFNγ markers. **A** and **B**. Representative histograms show the frequency of CD4^+^ and CD8^+^ T cells (gated from lymphocyte population) and IFNγ-positive CD4^+^ or CD8^+^ T cells following treatment with S peptides in lungs (**A**) or spleen (**B**). **C** and **D** Frequency and absolute numbers of T cells per 10^6^ total lung cells (**C**) or spleen cells (**D**). **E** IFNγ secretion by lung and spleen cells at 28 days post vaccination. Cells were isolated from tissue samples of uninfected animals and stimulated with SARS-CoV-2 S-peptide pool. The level of IFNγ was determined in supernatants of cultured cells by ELISA. Data represent mean ± SEM of 4–5 animals per group. *p < 0.05; **p < 0.01; ***p < 0.001; ****p < 0.0001 (one-way ANOVA with multiple comparisons, Fisher’s LSD test).

The immune cells isolated from organs were stimulated with a pool of SARS-CoV-2 S peptides, and IFNγ^+^ T-cells were quantified by flow cytometry (Fig. 4A–D, Supplementary Fig. 4). Significantly higher percentages and the total numbers of IFNγ^+^CD4^+^ T cells and IFNγ^+^CD8^+^ T cells in the lungs and spleen were detected in hamsters vaccinated with HPIV3/full S compared to all other groups. The levels of IFNγ secreted in the culture supernatants following peptide treatment were quantified by ELISA. Significantly greater levels of IFNγ were detected in supernatants of the lung cells in HPIV3/full S group compared to all other groups (Fig. 4E, left panel). Although the magnitude of IFNγ levels secreted by spleen cells was lower than that of lung cells, HPIV3/full S vaccine induced secretion of greater levels of IFNγ compared to the other constructs (Fig. 4E, right panel). These results demonstrate that HPIV3/full S induces robust memory Th1 and CD8^+^ T cell responses in lungs and spleen.

### The HPIV3 full S vaccine construct protects hamsters from SARS-CoV-2 infection and disease

**Fig. 5:**
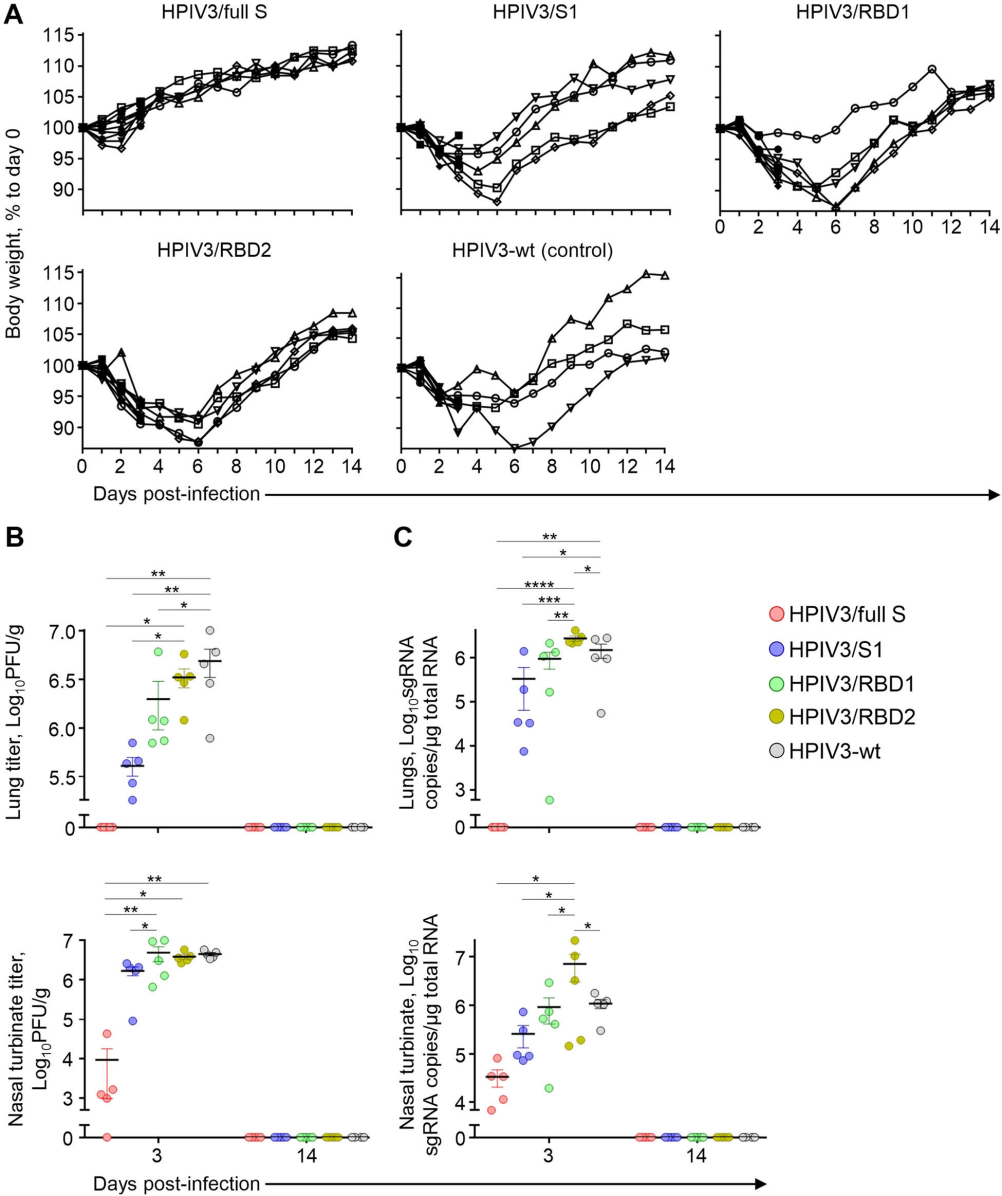
HPIV3 vaccine expressing full S protein protects hamsters against SARS-CoV-2 infection. Groups of hamsters at 10 animals per group were immunized with the indicated vaccine constructs by the intranasal route and challenged with SARS-CoV-2 in 28 days after immunization. Body weight curves are shown (**A**). On days 3 or 14 post-infection, animals were euthanized to determine viremia (**B)** and viral load (**C**) in lungs and nasal turbinates. **B**, **C** each dot corresponds to individual sample. Data represent mean ± SEM of *n* = 4–5 per group. *p < 0.05; **p < 0.01; ***p < 0.001; ****p < 0.0001 (one-way ANOVA with multiple comparisons, Fisher’s LSD test).

Four weeks after vaccination, hamsters were challenged by the IN route with 10^5^ PFU of SARS-CoV-2. Consistent with the immune response data, HPIV3/full S completely prevented the reduction of body weight upon infection. Animals from the other groups demonstrated weight loss reaching a maximum of approximately 10% on days 5–6 post-infection (dpi) (Fig. 5A). The hamsters were serially euthanized at 3 and 14 dpi to determine viral load (Fig. 5B) and viral RNA level (Fig. 5C) in tissues. Strikingly, no virus was detected in the lungs of HPIV3/full S immunized animals at 3 dpi by either plaque assay or qRT-PCR method. The HPIV3/S1 and HPIV3/RBD1 constructs also reduced the virus load in the lungs compared to the HPIV3-wt control at 3 dpi. Only HPIV3/full S construct significantly reduced viral titers in nasal turbinates when compared to other groups. At 14 dpi, SARS-CoV-2 was not detected in any animal, suggesting the resolution of active viral replication by this time point.

**Fig. 6:**
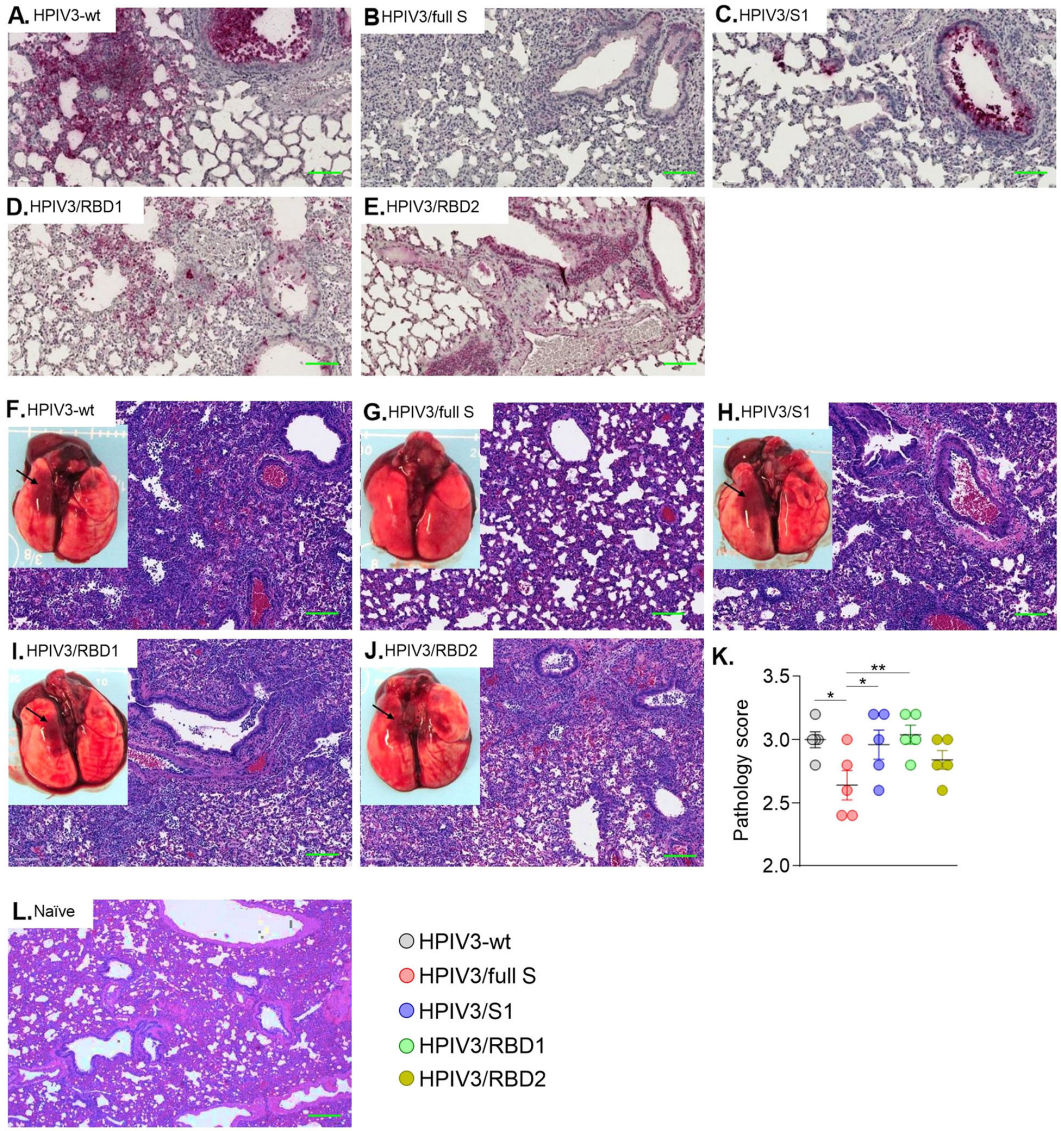
Virus-induced lung pathology in vaccinated hamsters on day 3 after infection. Immunohistochemical staining of SARS-CoV-2 nucleoprotein (N) antigen in lung tissues (**A**–**E**) and representative gross and histological lung images (**F–J**). Black arrows show congestion and focal consolidation in the lungs of SARS-CoV-2 infected hamsters. Magnification: ×4. **K** comparative pathology scores calculated based on criteria described in Supplementary Table 3. Data represent mean ± SEM of *n* = 5 per group. *p < 0.05; **p < 0.01 (one-way ANOVA with multiple comparisons, Fisher’s LSD test). **L** histological lung image of naïve hamster taken from our previous study^83^ for comparison. Scale bar: 0.25 µm.

Immunohistochemical staining for viral antigen (N) in lungs revealed mild to severe immunostaining pattern among different groups (Fig. 6A–E, Supplementary Fig. 5A–E; Supplementary Table 2). At 3 dpi, a moderate to severe immunostaining was observed in all groups except HPIV3/full S which showed only barely detectable staining at a few places (Fig. 6A–E), suggesting low-level transient replication of the virus. At 14 dpi, no-to-mild level of immunostaining was observed in all groups except for animals vaccinated with HPIV3/RBD1 and HPIV3/RBD2 which had mild-to-moderate staining (Supplementary Fig. 5A–E). These data are consistent with the viral load determined by plaque assay and qRT-PCR (Fig. 5B, C). On gross examination of the lungs, a generalized congestion and consolidatory foci were seen in the empty vector-vaccinated control animals euthanized at 3 dpi (Fig. 6F) or 14 dpi (Supplementary Fig. 5F). These gross changes were absent or minimal in hamsters vaccinated with the HPIV3/full S construct (Fig. 6G, Supplementary Fig. 5G), but the other groups had gross lesions comparable to that in the control animals (Fig. 6H–J, Supplementary Fig. 5H–J). At 3 dpi, typical interstitial pneumonia was noticed in lungs from the vector-vaccinated control animals which was characterized by a severe inflammation with mononuclear cells and heterophils in the alveolar space, interstitial septa and airways septal thickening, perivascular cuffing and vascular endothelial cell damage (Fig. 6F). In contrast, in animals vaccinated with the HPIV3/full S construct, moderate inflammation with lesser vascular and airway changes and lesser numbers of inflammatory macrophages and heterophils were observed (Fig. 6G,K). Furthermore, in animals vaccinated with the HPIV3/S1 (Fig. 6H), HPIV3/RBD1 (Fig. 6I) or HPIV3/RBD2 (Fig. 6J) constructs, a moderate to severe interstitial pneumonic changes that are comparable to vector-vaccinated control animals were detected. On day 14, an interstitial pneumonia with marked septal thickening was prominent in vector-vaccinated control (Supplementary Fig. 5F), as well as in HPIV3/S1 (Supplementary Fig. 5H), HPIV3/RBD1 (Supplementary Fig. 5I) and HPIV3/RBD2 groups (Supplementary Fig. 5J). However, the inflammatory changes were mild-to-moderate in HPIV3/full S group (Supplementary Fig. 5G,K), but a moderate septal thickening was still present in this group. In summary, vaccination with HPIV3/full S limits severe lung pathology and reduces the inflammatory changes. Thus, IN administration of a single dose of HPIV3/full S elicited protection in the upper respiratory tract and the lungs.

### SARS-CoV-2 challenge of animals vaccinated with the full S vaccine construct induces Th1 environment in the lungs

We tested the effects of the vaccination on cytokine profile in the lungs post-challenge. The cytokine levels in lung homogenates were analyzed by ELISA. At 3 dpi, the levels of IL-2, IL-4, IL-6, IL-10 and TNFα were similar in all groups, except from the slight IL-6 reduction by HPIV3/RBD2 compared to the control group (Supplementary Fig. 6A). However, the level of IL-12 was significantly higher in the HPIV3/full S group when compared to all other groups. Although the level of IFNγ was higher in HPIV3/full S than in HPIV3/RBD2 or vector-control group, it was not significantly different from HPIV3/S1 or HPIV3/RBD1 group. Interestingly, on day 14 post-infection, the levels of IL-12 and IFNγ were significantly higher in HPIV3/full S group compared to all other groups (Supplementary Fig. 6B). At this time, there were no differences in IL-2 or IL-4 levels between HPIV3/full S and the control group, and IL-10 secretion remained similar in all groups. The IL-6 levels were reduced in all vaccinated animals compared to control animals. The decrease in TNFα secretion levels was observed in HPIV3/full S and HPIV3/RBD1 groups. Our data demonstrate that SARS-CoV-2 challenge of HPIV3/full S vaccinated animals triggers Th1-type environment in the lungs, which is associated with protection.

### Vaccination with HPIV3 vaccine expressing the full S protein prevents inflammatory response in the lungs upon challenge

**Fig. 7:**
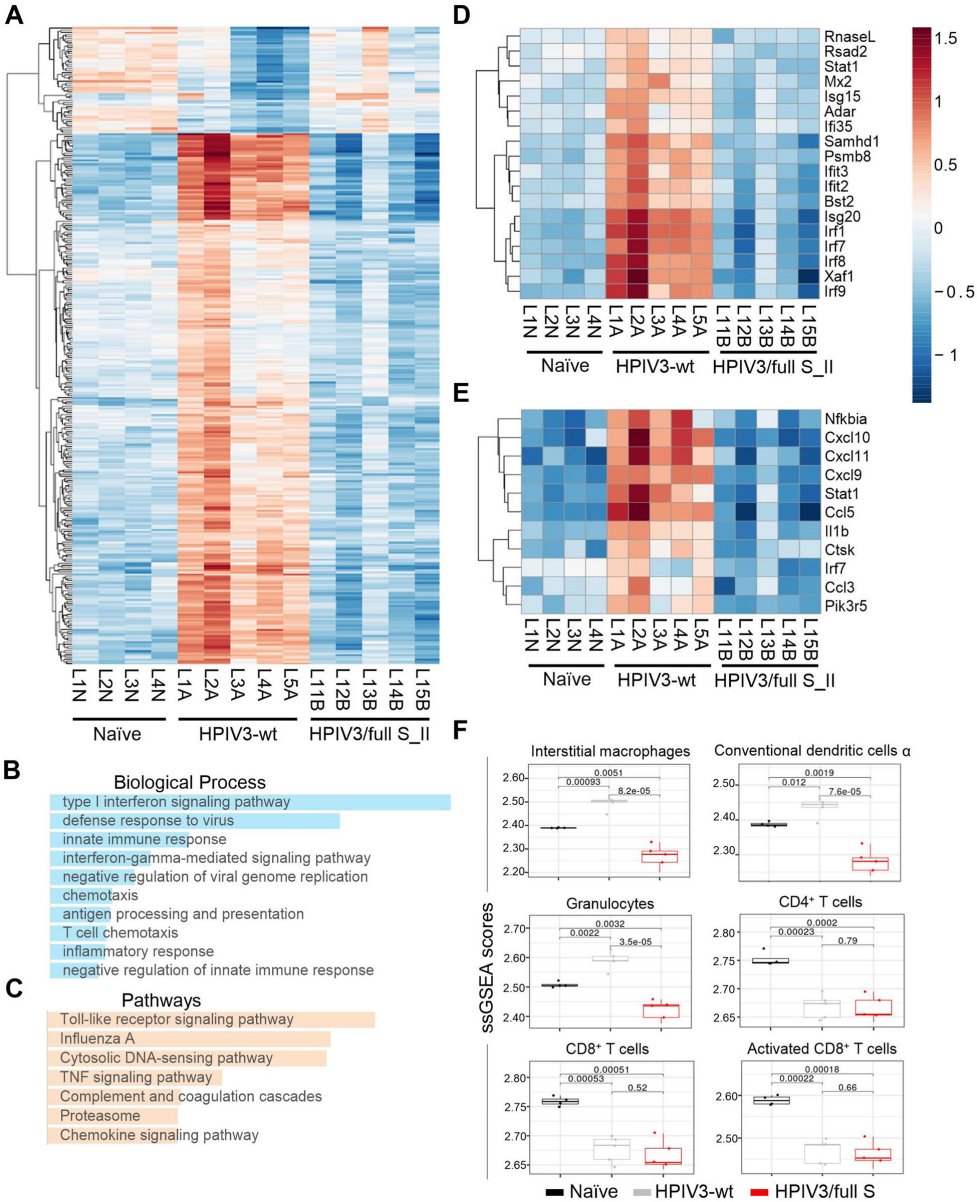
Gene expression pattern in vaccinated hamsters at 3 dpi resembles that of naïve animals. RNA seq of lung tissue harvested from naïve animals (unvaccinated, uninfected), or at 3 dpi from HPIV3-wt or HPIV3/full S vaccinated hamsters, was performed. **A** Heat map of the top 300 differentially expressed genes with hierarchical clustering of genes is shown at the left. The color legend indicates log_2_ expression measures. **B**, **C** Gene ontology enrichment analysis of differentially expressed genes upregulated in lungs of HPIV3-wt hamsters relative to HPIV3/full S vaccinated hamsters. **D**, **E** Heat map of top enriched GO category type I interferon signaling pathway (**D**) and toll-like receptor signaling pathway (**E**)**. F** Cell-type proportions of bulk RNA sequencing data. To evaluate changes in lung cellular composition across the animal groups, the single sample gene-set enrichment analysis (ssGSEA) was applied to quantify the relative abundance of different cell types across samples. ssGSEA scores were tested for differences between sample groups with a two-tailed *T*-test.

To characterize the global transcriptome response in lungs of vaccinated hamsters after the challenge, we performed RNAseq at 3 dpi comparing the HPIV3/full S group (*n* = 5) to the HPIV3-wt control group (*n* = 5). Lung samples from naïve hamsters (mock-infected with media, *n* = 4) served as a baseline control for analysis. Heat map of the top 300 differentially expressed genes revealed a significant upregulation of 250 genes in HPIV3-wt group relative to the naïve group, which included several chemokines and cytokines (Fig. 7), the data consistent with induction of inflammation and cytokine storm in COVID-19 patients^36,37^. In contrast, HPIV3/full S-vaccinated animals demonstrated a gene expression pattern closely resembling that in the naïve group, with a striking downregulation of multiple genes which contribute to the inflammation. Gene ontology analysis of the genes upregulated in HPIV3-wt group revealed a significant enrichment of several biological processes characteristic of an acute viral infection (Fig. 7B, D), including IFN signaling, innate immune response, chemotaxis and inflammatory response. Similarly, pathway analysis revealed a significant enrichment of TLR, TNF, and chemokine signaling pathways (Fig. 7C, E). Chemokine gene expression heat map showed a marked reduction of chemokine expression in vaccinated hamsters (Supplementary Fig. 7A) when compared to upregulation in the HPIV3-wt group (*Cxcl9*, *Cxcl10*, *Cxc11*, and *Cxcl16*). Furthermore, we also observed differential expressed genes in HPIV3-wt versus naïve group (676 upregulated and 880 downregulated) and HPIV3/full S vaccinated versus naïve group (723 upregulated and 361 downregulated).

To gain biological insights of the gene expression profiling data, deconvolution of the bulk RNAseq data was performed using pathway-level information extractor (PLIER)^38^ to identify differences in cell-type proportion between the three groups. We detected a significant increase in the expression pattern associated with myeloid cell populations: interstitial macrophages, activated DC (DCα) and granulocytes in the mock-vaccinated infected animals compared to the naïve animals (Fig. 7F), suggesting a preponderance of acute inflammation. Vaccination with HPIV3/full S completely or partially prevented the infiltration of these cells, as well as alveolar macrophages, conventional DC (cDC), plasmacytoid DC (pDC) and monocytes (Supplementary Fig. 7B). We also detected a significant reduction of lymphoid cell populations: natural killer (NK) cells, CD4^+^ T cells, CD8^+^ T cells, activated CD8^+^ T cells and B cells, as well as in stromal cells, bronchiolar epithelial cells and pneumocytes in mock-vaccinated animals, but vaccination did not alleviate these effects. Additionally, gene set enrichment analysis of the entire dataset was performed using GAGE pathway analysis^39^ to identify enriched pathway categories, as even small coordinated gene expression changes in a pathway can lead to a greater biological effect. This identified COVID-19 (Supplementary Fig. 8A) and cytokine-cytokine receptor interaction (Supplementary Fig. 8B) pathways amongst the top enriched pathways. Thus, transcriptome profiling suggests that vaccination prevented inflammatory responses after the challenge.

## Discussion

Here, we developed a panel of HPIV3-based constructs expressing the SARS-CoV-2 full-length spike glycoprotein or its fragments and evaluated them as single-dose IN vaccines in the golden Syrian hamster model of COVID-19. The data demonstrated strong SARS-CoV-2 neutralizing antibody and T cell responses induced by the full S construct. Although RBD is the immunodominant region eliciting SARS-CoV-2-neutralizing antibodies^40,41^, and that the vast majority (~90%) of neutralizing antibodies detected after a natural human infection bind to RBD^11^, only the full S vaccine, but not other constructs expressing the S1 protein subunit or any of the two versions of RBD, was able to induce strong immune responses that protected animals against the infection. However, membrane-anchored form of the S1 subunit expressed by a replication-competent vesicular stomatitis virus (VSV)^42^ or inactivated rabies virus vectored vaccine^43^ induced virus-neutralizing antibody response and protected animals from SARS-CoV-2 challenge. Furthermore, the transmembrane RBD presented as a chimeric minispike in VSV replicon^44^, virus spike RBD fused together as a tandem repeat^45^, or RBD trimerized via fusion to the trimerization domain of T4^46^ were also shown to elicit neutralizing antibodies in BALB/c mice. Thus, the membrane anchored or oligomerized forms of RBD displayed by these vaccines could have better preserved the conformation of B cell epitopes and thereby immunogenicity against S1 or RBD, compared to the S1 and RBD constructs in this study.

Despite good efficacy of multiple IM vaccines against SARS-CoV-2, a direct mucosal delivery may be necessary to attain robust protective immunity in the lungs. In BALB/c mice, only an IN inoculation, but not an IM injection, of a replication-incompetent recombinant adenovirus serotype 5 that carries the full-length SARS-CoV-2 S protein (Ad5-S-nb2) was able to elicit neutralizing antibody response in the bronchoalveolar lavage fluid^47^. In the study with an integrase-deficient, non-integrative version of lentivirus expressing the full-length S protein administered as IM prime/IN boost vaccine, the boost immunization significantly improved protection against virus replication in lungs of the SARS-CoV-2-infected hamsters, even though it did not improve the serum neutralizing activity. Nevertheless, the complete protection was still not achieved^48^. In another study, an IN vaccination with a chimpanzee adenovirus (simian Ad-36) encoding the S protein induced higher serum neutralizing titers, better protected hamsters, and was more potent in reduction of viral load when compared to IM vaccination^49^. However, even an IN vaccination with the construct was unable to completely prevent virus replication in the lungs of SARS-CoV-2-infected hamsters^49^ or rhesus macaques^50^. In our study, a single dose of HPIV3/full S provided excellent protection of the lower respiratory tract against virus replication, as evidenced by the lack of any detectable infectious virus or viral RNA at 3 dpi in our study. Furthermore, very limited pathologic changes and mild antigen staining were observed in the lungs of hamsters vaccinated with the full S construct. Given the extremely high susceptibility of hamsters to SARS-CoV-2, the abrogation of virus replication in hamsters vaccinated with the full S construct is presumably attributed to effective neutralization of the virus by vaccine-induced antibodies at the mucosal surfaces. Consistently, HPIV3/full S vaccine reduced replication of virus in nasal turbinates to barely detectable levels. Even with IN route of vaccine delivery, it is difficult to achieve complete protection against SARS-CoV-2 in the upper respiratory tract^47,49–53^. When high concentrations of potent RBD-specific neutralizing antibodies were applied intranasally 12 h prior to challenge, the robust SARS-CoV-2 infection was still seen at 4 dpi in nasal turbinates, but not in the lungs of hamsters^54^. However, in two studies using the ferret animal model, full protection of the upper respiratory tract was observed after immunization (simultaneous oral and IN delivery) with a replication-deficient adenovirus serotype 5 encoding the spike protein (Ad5-nCoV)^55^, or IN immunization with parainfluenza virus 5 expressing the S protein^56^, although in the latter study virus transmission from vaccinated to naïve animals still occurred. In the first reported ferret challenge experiment^57^, viral shedding was mainly observed in the upper respiratory tract, but infectious viral titers were low. The differences in disease severity among the animal models used to test SARS-CoV-2 vaccines, with the ferret model reproducing subclinical or mild human infections and the hamster model recapitulating lung pathology seen in patients with severe COVID-19^58^, complicates the direct comparison of vaccine candidates.

Using a peptide microarray, we identified linear S protein epitopes targeted by vaccine-induced serum IgG. Five major linear epitopes have been identified, with four of them located outside RBD. The epitope found within RBD was outside of the receptor-binding motif, and antibodies to this epitope were detected in only one out of 10 pre-challenge serum samples from vaccine-immunized hamsters, with one of the lowest neutralizing titers across the group (Supplementary Table 1). Of note, early B cell response to the S protein analyzed from a COVID-19 patient was polyclonal and at epitopes mostly outside of the RBD^59^. The linear epitope landscape of the SARS-CoV-2 spike protein constructed from 1051 COVID-19 patients does not contain any relevant RBD epitopes^60^, suggesting that most of the epitopes in RBD are conformational. Epitope II in a close proximity to the S1/S2 cleavage site (669–683 aa) identified in our study (Supplementary Fig. 1) overlaps with S1-111 (661–672 aa) and S1-113 (673–684 aa) epitopes from the epitope landscape^60^. Presumably, antibodies targeting this epitope can interfere with S1/S2 cleavage during the S protein processing, thus contributing the neutralizing serum activity. Interestingly, the antibody which blocks cleavage of Ebola virus (EBOV) GP into GP1 and GP2 subunits^61^ was shown protective in a non-human primate model against otherwise lethal EBOV infection^62^. Epitope III which covers the S2′ cleavage site and fusion peptide (813–827 aa) overlaps with S2-22 (812–823 aa), S2-23 (818–829 aa)^60^, and S21P2 (809–826 aa) epitopes^63^ identified when analyzing sera from COVID-19 patients. The antibodies against this epitope can neutralize SARS-CoV-2^63^, likely by blocking the cleavage and disturbing the function of FP. The cleavage at S2′ site is an important step to prime the S protein for membrane fusion. An inhibitor of TMPRSS2, serine protease which can mediate this cleavage, blocks SARS-CoV-2 infection of lung cells^64^. Most of the analyzed pre-challenge serum samples from hamsters vaccinated with HPIV3/full S construct (7 out of 10) contained antibodies with binding sites located next to HR1 (Supplementary Table 1). The antibodies targeting HR1/2 may block the conformational changes that are essential for effective virus-cell fusion^65^. Interestingly, antibodies that recognize parts of the viral envelope responsible for membrane fusion have been also identified for other viruses. Thus, 5 out of 6 most potent broadly neutralizing ebolavirus antibodies were shown to contain epitopes in the internal fusion loop of GP^66^. Human monoclonal antibody BDBV259 binding to internal fusion loop of the Bundibugyo ebolavirus GP was shown to inhibit virus cell entry^67^. Overall, the epitopes found in S2 subunit are highly conserved across virus variants (Supplementary Fig. 1), and likely contribute neutralization of WA1/2020 and VOCs (Fig. 2B–D) by interfering with viral fusion machinery.

Although antibody response is important for neutralization of virus, the involvement of cell-mediated responses in SARS-CoV-2 clearance cannot be underestimated^68^. HPIV3-vectored vaccine induced both CD8^+^ T cell and CD4^+^ Th1 memory cell responses in the lung tissues. The excessive Th2 cytokine-biased responses and inadequate Th1-biased T cell response were shown to contribute the immunopathology during SARS-CoV infection in animal models^69–71^. Induction of effective CD8^+^ T-cell response in the lungs by the vaccine has two important advantages. First, CD8^+^ T-cells provide additional protection against infection at the portal of entry. Second, mutations in the S protein of SARS-CoV-2 variants emerge as a result of selective pressure of antibodies^3^ rather than cytotoxic T cells, and therefore are unlikely to significantly affect sensitivity of CD8^+^ T cell epitopes to cell-mediated response. As such, the antiviral effect of cytotoxic T cell response is likely to equally contribute to protection against both WA1/2020 and VOCs.

Vaccination with HPIV3/full S elicited increase in both IFNγ and IL-12 following SARS-CoV-2 challenge (Supplementary Fig. 6). Both IFNγ and IL-12 activate macrophages and NK cells and increase antiviral activity of other tissue cells by upregulation of MHC class I/II molecules^72^. On the other hand, vaccination limited the secretion of the proinflammatory cytokines TNFα and IL-6, which are involved in the acute inflammatory response, after the challenge. Supporting this, patients with severe COVID-19 had markedly higher levels of IL-6 and TNFα pro-inflammatory cytokines compared to patients with mild-to-moderate disease^36,37^. Furthermore, the bulk transcriptome analysis confirmed that HPIV3/full S vaccine prevented an overexpression of multiple chemokines and cytokines (Fig. 7, Supplementary Fig. 7A) and subsequent influx of inflammatory cells including granulocytes and interstitial macrophages (Supplementary Fig. 7B), which is a hallmark of COVID-19 pathogenesis^36,37^. Consistently, the histopathological analysis revealed a reduction in overall pneumonic changes including the extent of cellular infiltration and changes in vascular and airway compartments. Importantly, the presence of interstitial macrophages and granulocytes was lower in lungs of animals that were vaccinated with HPIV3/full S construct. The interstitial macrophages are attributed for excessive inflammatory response and tissue pathology^73,74^. Finally, no signs of increased pathology due to potential antibody-dependent enhancement were observed in vaccinated animals. Nevertheless, further investigation of this vaccine in additional animal models is required prior to considering clinical trials.

Essentially all humans are infected with HPIV3 during early childhood. Therefore, there is a concern that efficacy of HPIV3-vectored vaccines in adults might be reduced due to the prevalence of preexisting immunity against HPIV3 acquired by natural exposure^26^. We demonstrated that HPIV3/full S vaccine construct induces less HPIV3-neutralizing antibodies compared to the vector control (Fig. 2E). Moreover, in guinea pigs that had previously been infected with HPIV3, immunization with a single dose of HPIV3 expressing EBOV GP (HPIV3/EboGP) induced titers of EBOV-specific serum antibodies that nearly equaled those detected in HPIV3-naïve animals^75^. HPIV3/EboGP was also shown to replicate, although at a reduced level, in the respiratory tract of rhesus macaques, despite the preexisting immunity to vaccine vector, and elicit EBOV-specific neutralizing response. Remarkably, the neutralizing titers were even higher in HPIV3-immune monkeys after two vaccine doses each administered by the combined IN and intratracheal route, compared to HPIV3-naïve animals^76^. Further studies are required to determine the effects of HPIV3 preexisting immunity on protection elicited by HPIV3-vectored vaccines. However, in any case, HPIV3/full S could be a good candidate for a pediatric needle-free bivalent vaccine against SARS-CoV-2 and HPIV3, as it is based on the JS strain of HPIV3 which is expected to be naturally attenuated in humans^27^.

Given an unprecedented global spread of SARS-CoV-2 and emergence of the new virus variants resistant to neutralization, protection induced by CD8^+^ T cells in the lungs induced by the vaccine is unlikely to be significantly reduced by mutations present in the current and future SARS-CoV-2 variants. Although not tested here, we anticipate that the second dose of HPIV3/full S would augment the antibody response, based on our previous study with an HPIV3-vectored vaccine against Ebola virus^77^. In addition, HPIV3 vector platforms may be considered for boost IN vaccination following IM prime with currently approved vaccines to stimulate balanced systemic and local immune response in the respiratory tract. The data obtained in our study suggest HPIV3/full S is a promising vaccine candidate and justify its preclinical evaluation.

## Methods

### Viruses

The SARS-CoV-2 strain used for animal challenge is the first US isolate SARS-CoV-2 USA_WA1/2020 from the Washington State patient identified on January 22, 2020 (GenBank accession number: MN985325)^78^. Passage 3 was obtained from the World Reference Center for Emerging Viruses and Arboviruses (WRCEVA) at UTMB and underwent two more passages on Vero-E6 cells (passage 5). For virus neutralization assay, viral stock which underwent two additional passages, was used (passage 7). Alpha (lineage B.1.1.7, isolate SARS-CoV-2/human/USA/CA_CDC_5574/2020), Beta (lineage B.1.351, isolate hCoV-19/USA/MD-HP01542/2021) and Gamma (lineage P.1, isolate SARS-CoV-2/human/USA/MD-MDH-0841/2021; GenBank accession number: MW621433) variants of SARS-CoV-2 were obtained from WRCEVA, passage 2 (Gamma) or 3 (Alpha, Beta), and subjected to an additional passage on Vero-E6 cells. The inoculum stocks used in virus neutralization assay were passages 3 or 4, respectively. The recombinant SARS-CoV-2 expressing mNeonGreen reporter—WA1/2020^31^, Kappa (lineage B.1.617.1) and Delta (lineage B.1.617.2) variants—were kindly provided by Dr. Pei-Yong Shi (UTMB). For all SARS-CoV-2 variants, S ORF was sequenced in viral stocks used for in vitro experiments. The following mutations were identified: Gamma, K417T; Delta, deletion of amino acids 689–691. No other changes were found compared to the corresponding reference sequences. All studies involving infectious SARS-CoV-2 were performed under BSL-3 containment of the Galveston National Laboratory (GNL), UTMB. HPIV3 wild-type (wt), strain JS^79^ was used as vehicle control in vaccination experiments.

### Generation of vaccine constructs

HPIV3-vectored vaccine constructs expressing SARS-CoV-2 full S protein (1–1273 aa), its S1 subunit (1–685 aa), RBD1 (319–591 aa) or RBD2 (319–529 aa) were generated (the amino acid sequences of S protein are based on the first US SARS-CoV-2 isolate, GenBank accession no. MN985325). The vaccine vector was based on HPIV3 SUDV GP full-length clone^80^ generated with HPIV3 reverse genetics system^81^ kindly provided by Peter L. Collins (NIAID, NIH). For optimal immunogen expression in mammalian cells, different strategies for codon optimization in S ORF were utilized, resulting in the two sets of constructs. Coding sequences for full S, S1, RBD1 or RBD2 flanked by NcoI and BlpI restriction sites were PCR-amplified with PfuUltra high-fidelity DNA polymerase (Agilent Technologies) using cDNA not codon-optimized for eucaryotic translation (set “a”) or codon-optimized (set “b”). The codon-optimized (Genscript) cDNA in pCR-Blunt II-TOPO vector was kindly provided by Dr. Matthias J. Schnell (Thomas Jefferson University). Because of the overlapping NcoI site with the ORF start (ATGG), the second amino acid in full S and S1 constructs was different from that in an original SARS-CoV-2 S sequence (P → V). For RBD constructs, Val codon (GTC or GTT) was introduced immediately downstream of the start codon, followed by sequences encoding for 319–591 aa or 319–529 aa. The reverse primers were designed to introduce stop-codons (TGA for HPIV3/full S_b, and TAA for other 7 constructs) followed by four-nucleotide TATA sequence to comply with the “rule of six” for paramyxovirus genomes^82^. Obtained PCR fragments were introduced into HPIV3 SUDV GP by NcoI and BlpI restriction sites to replace SUDV GP ORF. A complete nucleotide sequences of the resulting full-length clones were verified by Sanger sequencing. The full-length clones were next used to recover vaccine constructs as previously described^80^. Passage 0 aliquots were amplified in LLC-MK2 cells for 2–3 passages to raise the sufficient stocks for experiments. The inserts encoding for S protein or its fragments were sequenced in genomic RNA of all recovered viruses, with no mutations identified. To determine viral titers, viruses were inoculated onto LLC-MK2 monolayers, and incubated for 8 days at 32 °C, 5% CO_2_ under 0.45% methylcellulose (Sigma-Aldrich) overlay. Then, monolayers were fixed with ice-cold methanol, and viral plaques were immunostained with wiffleball fluid of HPIV3-infected rabbits provided by Drs. Peter Collins and Ursula Buchholz (NIAID) and HRP-labeled goat anti-rabbit IgG secondary antibody (ThermoFisher Scientific).

### Western blot

The expression of SARS-CoV-2 S proteins by infected cells was detected by Western blot as in^80^. Briefly, monolayers of LLC-MK2 cells were infected with HPIV3/full S_a, HPIV3/full S_b, HPIV3/S1_a, HPIV3/S1_b, HPIV3/RBD1_a, HPIV3/RBD1_b, HPIV3/RBD2_a, HPIV3/RBD2_b, HPIV3-wt, or mock-infected. After 48 h, cells were lysed and run on a 4 to 12% SDS-PAGE gel. The recombinant S protein (Sino Biological) was loaded at 2.5 µg as control. The membrane was stained with primary rabbit anti-SARS-CoV-2 S polyclonal (Sino Biological) and mouse actin pan monoclonal (ThermoFisher Scientific) antibodies, followed by secondary goat anti-rabbit IgG 800 CW and anti-mouse IgG 680 RD antibodies (LI-COR). Protein bands were visualized with a LI-COR Odyssey Fc imaging system.

### Hamster studies

Six- to seven-week-old female golden Syrian hamsters (*Mesocricetus auratus*) were obtained from Envigo. Hamsters were housed at 4 animals per isolator cage and provided food and water *ad libitum*. SARS-CoV-2 challenge study was conducted in an animal biosafety level 3 (ABSL-3) GNL facility. The animal protocol for testing of HPIV3-based vaccine constructs in hamsters was approved by the UTMB Institutional Animal Care and Use Committee in compliance with the Animal Welfare Act and other applicable federal statutes and regulations relating to animals and experiments involving animals. Five groups of hamsters (*n* = 10 per group) were intranasally vaccinated with HPIV3/full S, HPIV3/S1, HPIV3/RBD1, HPIV3/RBD2, or mock-vaccinated with HPIV3-wt, at 10^6^ PFU in a total volume of 100 µl (~50 μl into each nostril). Four weeks after the vaccination, animals were challenged with SARS-CoV-2 intranasally at 10^5^ PFU in a total volume of 100 µl administered as described above. Over the infection course, hamsters were monitored daily for weight changes. On each serial endpoint day (days 3 and 14 post-challenge), lungs, nasal turbinates and sera were collected from 5 hamsters per group. Additionally, serum samples were collected from all animals at one day prior to vaccination, and one day prior to challenge. One animal in HPIV3-wt group was found dead on day 15 after vaccination (13 days prior to challenge) with no clinical signs of disease and was therefore excluded from the subsequent analysis.

In a separate study conducted under ABSL-2 containment, four groups of hamsters (*n* = 5 per group) were intranasally vaccinated with HPIV3/full S, HPIV3/S1, HPIV3/RBD1, or mock-vaccinated with HPIV3-wt, at 1 × 10^6^ PFU in a total volume of 100 µl. Four weeks after the vaccination, animals were euthanized, lungs and spleen were collected, and subjected to an analysis for the specific immune cell populations by flow cytometry.

### Analysis of viremia

The right lung and nasal turbinates were homogenized in Leibovitz L-15 cell culture medium supplemented with 10% fetal bovine serum (FBS) and 1% Antibiotic–Antimycotic (ThermoFisher Scientific) using the TissueLyser II bead mill and 5 mm stainless steel beads (Qiagen) and briefly centrifuged (5 min, 2000 × *g*, 4 °C). The duplicate 10-fold serial dilutions of homogenates were prepared in MEM medium (Gibco) and adsorbed on Vero-E6 monolayers in 96-well plates for 1 h at 37 °C, 5% CO_2_. The virus inoculum was then replaced with 0.55% methylcellulose overlay. After 2 days, an overlay was removed, and cells were fixed with 10% formalin at 4 °C for 24 h. Fixed monolayers were stained with 10% formalin containing 0.25% crystal violet (ThermoFisher Scientific) at room temperature for 20 min and washed with water. Plaques were counted, and virus load per gram tissue was determined.

### RNA isolation

The aliquots of supernatants from lung and nasal turbinate homogenates were dissolved in TRIzol LS (Life Technologies). Total RNA was isolated using Direct-zol™ RNA Microprep kit (Zymo Research) with on-column DNAse digestion according to the manufacturer’s recommendations. The final RNA solutions were stored at −80 °C until used for qRT-PCR or transcriptome analysis.

### Analysis of viral load by qRT-PCR

Replicating viral RNA was determined in the lungs and nasal turbinates by measuring subgenomic SARS-CoV-2 E gene RNA by qRT-PCR in triplicates as described previously^83^. An Ultramer DNA oligo (IDT Bioservices) spanning the amplicon was used to generate standard curves to calculate the sgRNA copies per microgram of the total RNA.

### Total IgG ELISA

The 96-well microplates (Greiner Bio-One) were coated with 1 µg/mL SARS-CoV-2 S protein (Sino Biological) diluted in phosphate buffered saline (PBS). After overnight incubation at 4 °C, plates were washed 4 times with PBS with 0.05% Tween-20 and blocked with SuperBlock blocking buffer (ThermoFisher Scientific) for 2 h at 37 °C. After washing, the duplicate 5-fold serial dilutions of hamster serum, starting at 1:10, were added in 50 µl/well (assay diluent: PBS + 0.05% Tween-20 + 5% goat serum). Plates were incubated for 2 h at 37 °C and washed 4 times, and then HRP-conjugated goat anti-Syrian hamster IgG (Abcam) was added at 1:10,000 dilution in assay diluent. Plates were incubated for 1 h at 37 °C and washed 4 times, and then one-component SureBlue reserve TMB microwell peroxidase substrate (Sera Care) was used to detect bound antibody. After incubation at room temperature for 10 min, the reaction was stopped by adding TMB BlueSTOP solution (Sera Care), and the absorbance was measured at optical density (OD) 630 nm. Titers were determined using a four-parameter logistic curve fit in GraphPad Prism 6.07 software and defined as the reciprocal dilution at approximately OD_630nm_ = 0.075 (normalized to the pre-vaccination serum sample for each animal).

### Virus neutralization assays

A total of 200 PFU of mNeonGreen SARS-CoV-2 (WA1/2020, Kappa variant, or Delta variant) were incubated in duplicates with 2-fold serial dilutions of serum starting from the initial dilution of 1:20 for 1 h at 37 °C in MEM medium containing 2% FBS and 0.1% gentamicin sulfate (Corning). Virus-serum mixtures were then added to Vero-E6 monolayers in black polystyrene 96-well plates with clear bottoms and incubated at 37 °C, 5% CO_2_. Plates were read using the Cytation 7 Cell Imaging Multi-Mode Reader (BioTek) (EX 488 nm, EM 528 nm) at 48 h post-infection. The percentage of neutralization was determined by the following formula: 100−((*X*−*Z*)*100/(*Y*−*Z*)), where *X* represents MFI readout of sample monolayers, *Y* is the MFI of virus-infected monolayers with no serum added, and *Z* is the MFI of uninfected monolayers (background).

The selected serum samples were also tested in a standard plaque reduction neutralization assay against biological isolates of the WA1/2020 strain and Alpha, Beta and Gamma VOCs. A total of 100 PFU of SARS-CoV-2 were incubated in duplicates with 2-fold serial dilutions of serum starting from the initial dilution of 1:20 for 1 h at 37 °C in MEM medium containing 2% FBS and 0.1% gentamicin sulfate. Virus–serum mixtures were then added to Vero-E6 monolayers in 24-well plates and incubated for 1 h at 37 °C, 5% CO_2_. The virus inoculum was then replaced with 0.67% methylcellulose overlay. After 3 days, an overlay was removed, and cells were fixed with 10% formalin at 4 °C for 24 h. Fixed monolayers were stained with 10% formalin containing 0.25% crystal violet at room temperature for 20 min and washed with water.

Samples from HPIV3/full S and HPIV3-wt groups were additionally tested for neutralization of HPIV3-wt in a plaque reduction assay (4 randomly selected samples/group on day −1, and 9 samples/group on day 27 post-immunization). A total of 30 PFU of HPIV3-wt were incubated in duplicates with 2-fold serial dilutions of serum starting from the initial dilution of 1:20 for 1 h at 37 °C in Opti-MEM medium (Gibco) containing 10% FBS and 0.1% gentamicin sulfate. Virus–serum mixtures were then added to LLC-MK2 monolayers in 96-well plates and incubated for 1 h at 37 °C, 5% CO_2_. The virus inoculum was then replaced with 0.44% methylcellulose overlay. After 3 days of incubation at 32 °C, 5% CO_2_, an overlay was removed, and cells were fixed with ice-cold methanol at 4 °C for 20 min. Fixed monolayers were immunostained with rabbit polyclonal antibodies against HPIV3 as described above for titration of the vaccine constructs.

In all neutralization assays, IC_60_ values were calculated in GraphPad Prism 6.07 software.

### Peptide microarray

Glass slides with imprinted 316 overlapping 15 residue peptides corresponding to SARS-CoV-2 S sequence (protein ID: P0DTC2) were produced by JPT Peptide Technologies GmbH (Berlin, Germany). The first peptide corresponds to residues 1 to 15 and each successive peptide begins 4 residues downstream (5–19, 9–23, etc.). Post-challenge sera were virus-inactivated by γ-irradiation at 5 MRad prior to the analysis. Slides were incubated with serum samples diluted at 1:50 for 1 h at 30 °C followed by five washes in JPT washing buffer. Then, slides were incubated with 0.1 µg/mL Cy5-conjugated goat anti-Syrian hamster IgG secondary antibody (Abcam) followed by five washes in JPT washing buffer. After additional two washes in deionized water, the slides were dried by centrifugation. MFI was recorded for each spot on the GenePix 4000b at 650 V and analyzed by GenePix Pro 7. Post-vaccination serum samples from animal group treated with HPIV3/full S vaccine were analyzed in triplicates; for all other samples, single runs were performed. Pre-vaccination sera served as background control. The results were expressed as mean values of specific signals produced by post-vaccination sera minus background, for each individual peptide.

### Histopathology

Following euthanasia of hamsters with ketamine/xylazine injection, necropsy was performed, and lungs were inspected for gross lesions. A representative specimen of lungs (right lower lobe) was collected in 10% buffered formalin for histological examination. Formalin-fixed tissues were processed per a standard protocol for histological analysis, and 4 μm-thick sections were cut and stained with hematoxylin and eosin (HE). Lung sections were examined under light microscopy using an Olympus CX43 microscope for the extent of inflammation, size of inflammatory foci, and changes in alveoli, alveolar septa, airways, and blood vessels. The slides were imaged in a digital scanner (Leica Aperio LV1). The blinded tissue sections were semi-quantitatively scored for pathological lesions using the criteria described in Supplementary Table 3.

### Immunohistochemistry

SARS-CoV-2 antigen (N) was identified in situ on formalin-fixed paraffin-embedded tissue sections by immunohistochemical (IHC) staining. Briefly, paraffin-embedded lung specimens were serially sectioned (5 µm). A citric acid-based antigen unmasking solution (Vector Laboratories Inc.), pH = 6 was used for the 20 min antigen retrieval process using a microwave (BioTek EZ-Retriever, BioGenix) heat treatment. A primary antibody specific for SARS-CoV-2 nucleocapsid (GeneTex) followed by a secondary anti-rabbit IgG AP (Vector Laboratories Inc.) were used for the staining. Viral antigen was visualized with the Vector^®^ Red AP Substrate Kit (Vector Laboratories Inc.) and analyzed by light microscopy.

### Cytokine ELISA

The level of cytokines in culture supernatants or lung homogenates were quantified using Immunotag^®^ Hamster ELISA kits (G-Biosciences), following the manufacturer’s instructions. Briefly, 100 μl of the lung homogenates or culture supernatants (4-fold diluted in Assay diluent) were incubated in duplicate wells pre-coated with cytokine-specific capture antibodies for 90 min at 37 °C. After two washes, 100 μl of biotin-labeled detection antibodies were added for 60 min at 37 °C. HRP–streptavidin conjugate was used for immunodetection with TMB substrate. The absorbance at 450 nm was measured. A standard curve obtained for each cytokine with known standards was used for calculating the level of cytokines.

### Immunophenotyping of lung and spleen cells by flow cytometry

For immunophenotyping, single cell suspensions were prepared from the lungs and spleens of vaccinated and control hamsters. Briefly, the lungs were cut into small pieces and suspended in 1 ml digestion buffer containing 3 mg/ml collagenase type II and 40 U/ml rDNase I (Worthington Biochemical). Following digestion for 40 min at 37 °C, the lung cells were passed through 70 µm cell strainer and collected as pellet by centrifugation (250 × *g*, 10 min). Red blood cells were lysed with ACK lysis buffer (Lonza) and washed out with an excess of 0.5% BSA in PBS (PBS/BSA). Cells were further processed for gradient centrifugation using Histopaque-1077 (Sigma-Aldrich) to enrich immune cells, following standard procedure. The final cell pellet was resuspended in PBS/BSA and cells were counted in TC20^TM^ Automated Cell Counter (Bio-Rad Laboratories). The spleen samples were gently minced with syringe plunger on top of the cell strainer and red blood cells were lysed as above. The cells were resuspended in PBS/BSA buffer and counted.

The supply of antibodies specific to hamster immune cells and proteins is very limited. As such, we optimized cell staining using anti-mouse antibodies. A previous study has shown that several anti-mouse antibodies bind to hamster immune cells well^84^. We incubated hamster lung and spleen cells with Live/Dead Fixable Aqua stain (BioLegend) and monoclonal antibodies specific for mouse CD4-FITC (clone GK1.5) and CD8-PE (clone eBio341, Invitrogen) T cell markers for 30 min at 4 °C in the dark. As well, cells were stained separately with B220-BV421 (clone RA3-6B2) and CD19-PerCP (clone 4G7) for B cell markers. All these antibodies, except CD8-PE, were purchased from the BioLegend. The stained cells were washed thoroughly with PBS/BSA and fixed in 2% paraformaldehyde (PFA) overnight. Fixed cells were analyzed on an LSR Fortessa flow cytometer (BD Biosciences) and frequencies of each cell type were calculated using FlowJo software (v10.8.0_CL). The T cell flow cytometry gating strategy is shown in Supplementary Fig. 4.

### Cell culture, peptide treatment, flow cytometry and cytokine quantification

To test in vitro recall response of vaccine candidates, we cultured the lung and spleen immune cells in 96-well plates (5 × 10^5^ cells/well) using RPMI-10% FBS (Gibco) supplemented with 1% Penicillin–Streptomycin–Glutamine (Sigma-Aldrich). The cells were treated with peptides of SARS-CoV-2 spike protein (PepTivator peptides Prot_S and Prot_S1) obtained from Miltenyi Biotec. As per manufacturer, the average purity of peptides was >70% (HPLC). The peptides were dissolved per manufacturer’s directions in sterile water and used at a concentration of 1 mg/ml. The cells were stimulated with peptides for 14 h at 37 °C, 5% CO_2_, and Brefeldin A (1 mg/ml, Sigma-Aldrich) was added for 2 h prior to collection of cells and culture supernatants. As a positive control, cells were stimulated with PMA/Ionomycin (1 mg/ml, Sigma-Aldrich) and, as a negative control, cells were left untreated.

After peptide treatment, the cells were collected and centrifuged at 200 × *g* for 5 min. The culture supernatants were harvested and stored frozen at −80 °C for ELISA quantification of cytokines as described below. The cells were surface stained with CD4-FITC (clone GK1.5) and CD8-PE (clone eBio341) antibodies for 30 min. After washing with PBS/BSA, cells were fixed with 2% PFA, permeabilized using Intracellular Fixation & Permeabilization Buffer (BioLegend) and stained with IFNγ-APC (clone XMG1.2, BioLegend) for 30 min at 4 °C in the dark. Then, cells were washed, resuspended in PBS, and analyzed on an LSR Fortessa flow cytometer (BD Biosciences). The frequency of cell types and IFNγ expressions were calculated using FlowJo software (v10.8.0_CL).

### Library construction, high-throughput sequencing and transcriptome analysis

Libraries for deep sequencing were made using the Smart-3SEQ protocol^85^. Briefly, the first strand primer was annealed to 1 μl of sample RNA and extended with SMARTScribe reverse transcriptase (Clontech, Inc). Second strand synthesis was performed after the addition of the second strand primer, and adapter sequences with unique indexes were added with 15 cycles of PCR with NEBNext single index adapters (New England BioLabs). PCR products were purified with AMPure XP SPRI beads (Beckman Coulter Life Sciences), quantified, and pooled for sequencing on an Illumina NextSeq 550 High Output Flow Cell with the single-end 75 base protocol.

The Smart-3SEQ protocol adds a five base unique molecular identifier (UMI) and 3 Gs to the 5′ end of each sequence. These were removed from the reads and the UMI was added to the read name with the umi_homopolymer.py software provided by the Smart-3SEQ authors. Reads were aligned to the *Mesocricetus auratus* NCBI assembly GCF_000349665.1 using STAR version 2.7.5c^86^ using the parameters recommended by the software authors for the Encode consortium. FeatureCounts software^87^ was used to count reads per gene using the NCBI annotation release 102. The count table was used as an input into DESeq2^88^, and differential gene expression was estimated following the DESeq2 vignette provided with the software. Hierarchical clustering of the genes was done with heatmap program in R. Gene ontology enrichment analysis was performed with DAVID v6.8^89,90^. Gene set enrichment analysis was performed with the GAGE software^39^ and Pathview software^91^ was used to produce the KEGG pathway figures, both following the vignettes provided by the authors.

Hamster-specific gene lists for stromal and immune lung cells were taken from a published single cell study of hamster SARS-CoV-2 infection^83^. These gene sets were used to compute ssGSEA scores from log transformed and quantile normalized bulk RNA seq data using the “gsva” function with parameters method = “ssgsea” and ssgsea.norm = T.

### Statistical analysis

Statistical analyses and generation of graphs were performed using GraphPad Prism version 6.07 (GraphPad Software). One- or two-way ANOVA with multiple comparisons (Fisher’s LSD test) or a *T*-test were used for determination of statistical significance.

### Reporting summary

Further information on research design is available in the Nature Research Reporting Summary linked to this article.

## Supplementary information

Supplemental Materials

REPORTING SUMMARY

## Data Availability

The datasets generated during the current study are available from the corresponding author on reasonable request. RNA-sequencing data have been deposited in NCBI’s Gene Expression Omnibus and are accessible through the GEO Series accession number GSE193288.
