# Supplementary material for: A single intranasal dose of human parainfluenza virus type 3-vectored vaccine induces effective antibody and memory T cell response in the lungs and protects hamsters against SARS-CoV-2
Source: NPJ Vaccines. 2022 Apr 25;7:47. doi: 10.1038/s41541-022-00471-3 (PMC9038905; doi:10.1038/s41541-022-00471-3)
Supplement: Supplementary file 1 — Supplemental Materials [file 41541_2022_471_MOESM1_ESM.pdf]

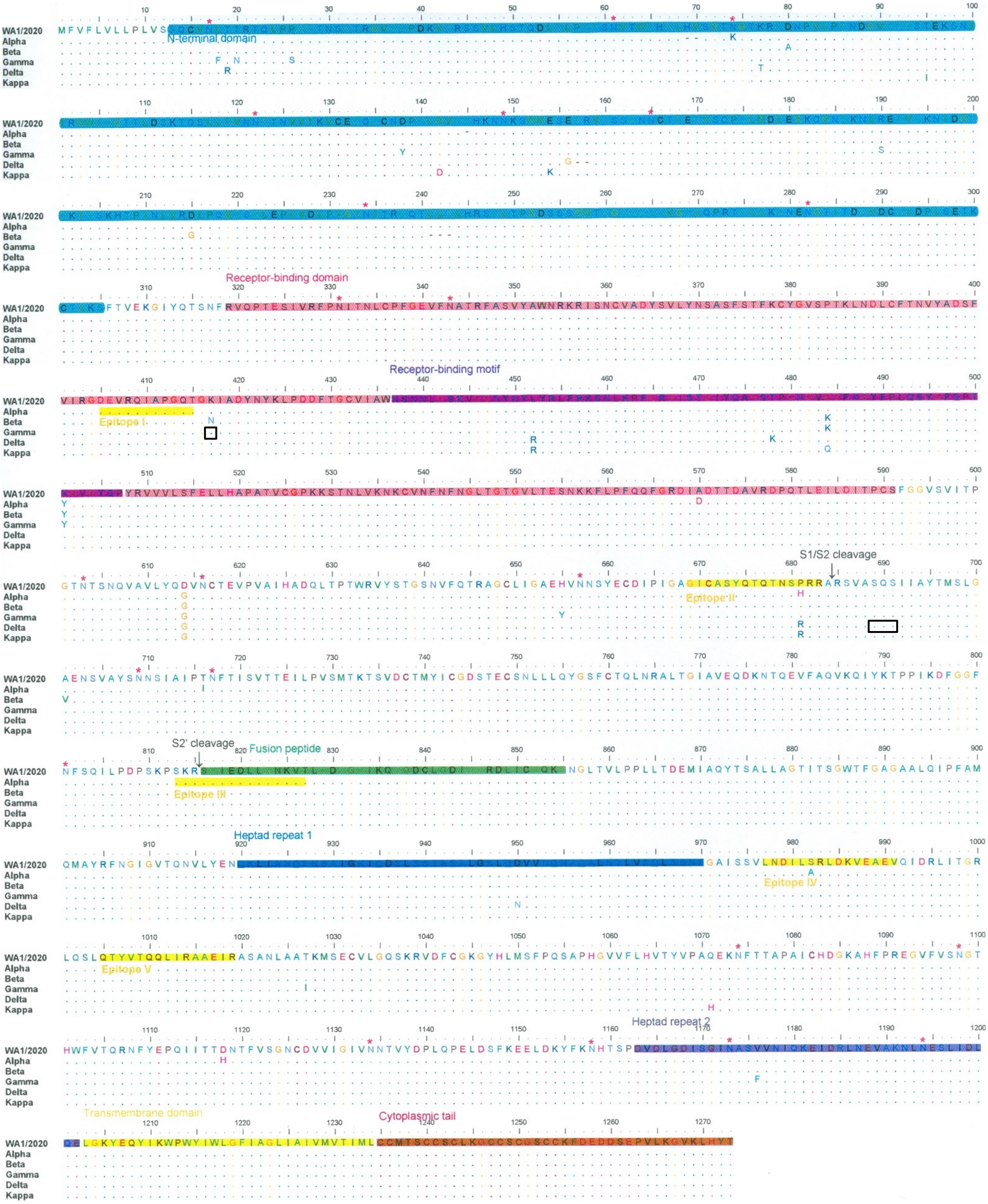

Supplementary Figure 1 (see next page for the legend).

Supplementary Figure 1 (related to Fig. 3)**. The spike protein alignment of major SARS-CoV-2 variants.** The GenBank accession numbers for sequences shown in alignment are as follows: WA1/2020 – QHO60594.1; Alpha – QUA12558.1; Beta – QRN78347.1; Gamma – QRV12072.1; Delta – QTW58946.1; Kappa – QTS25314.1. Epitope I: 405 – 415 aa (the overlapping part of peptides #101 and #102); epitope II: 669 – 683 aa (peptide #168); epitope III: 813 – 827 aa (peptide #204); epitope IV: 977 – 991 aa (peptide #245); epitope V: 1005 – 1019 aa (peptide #252). Stars indicate the N-linked glycosylation sites. Shown in boxes are the sites of mutations identified in viral stocks of Gamma and Delta variants used for in vitro experiments (see Materials and methods).

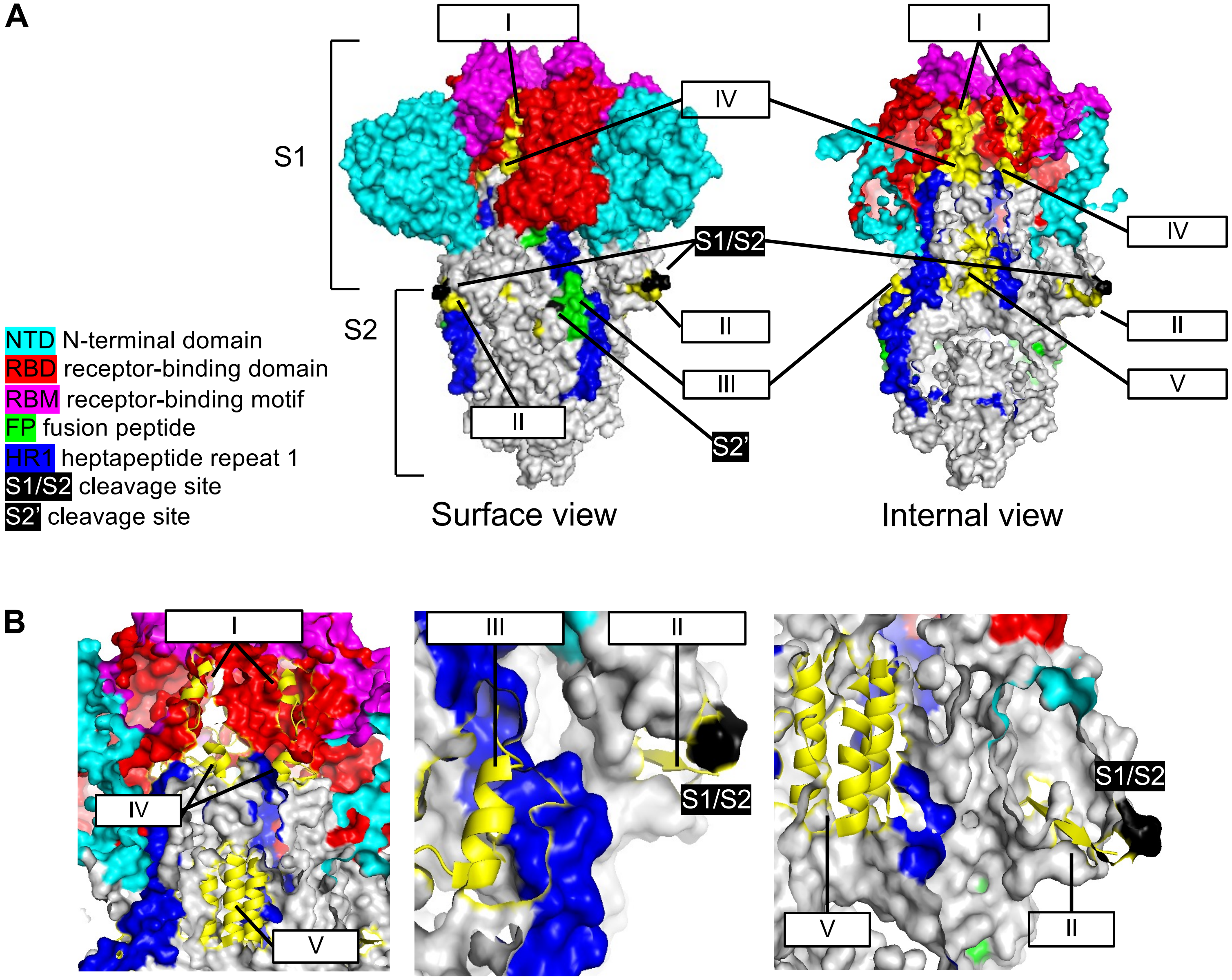

Supplementary Figure 2 (related to Fig. 3)**. The putative linear epitopes of vaccine-induced antibodies identified in peptide microarray.** The epitope I – V coordinates are given in Supplementary Figure 1. **A.** Five epitopes (yellow) mapped to SARS-CoV-2 S protein (PDB ID: 6ZP0) (Xiong et al., 2020). **B.** Structure and location of each epitope shown individually. The secondary structures of epitopes I, III, IV and V are presented by α-helices; epitope II – β-sheet. The structure simulation was based on the SARS-CoV-2 S protein (PDB ID: 6ZP0) using PyMOL Graphic software (Version 2.5.1, Schrödinger, LLC).

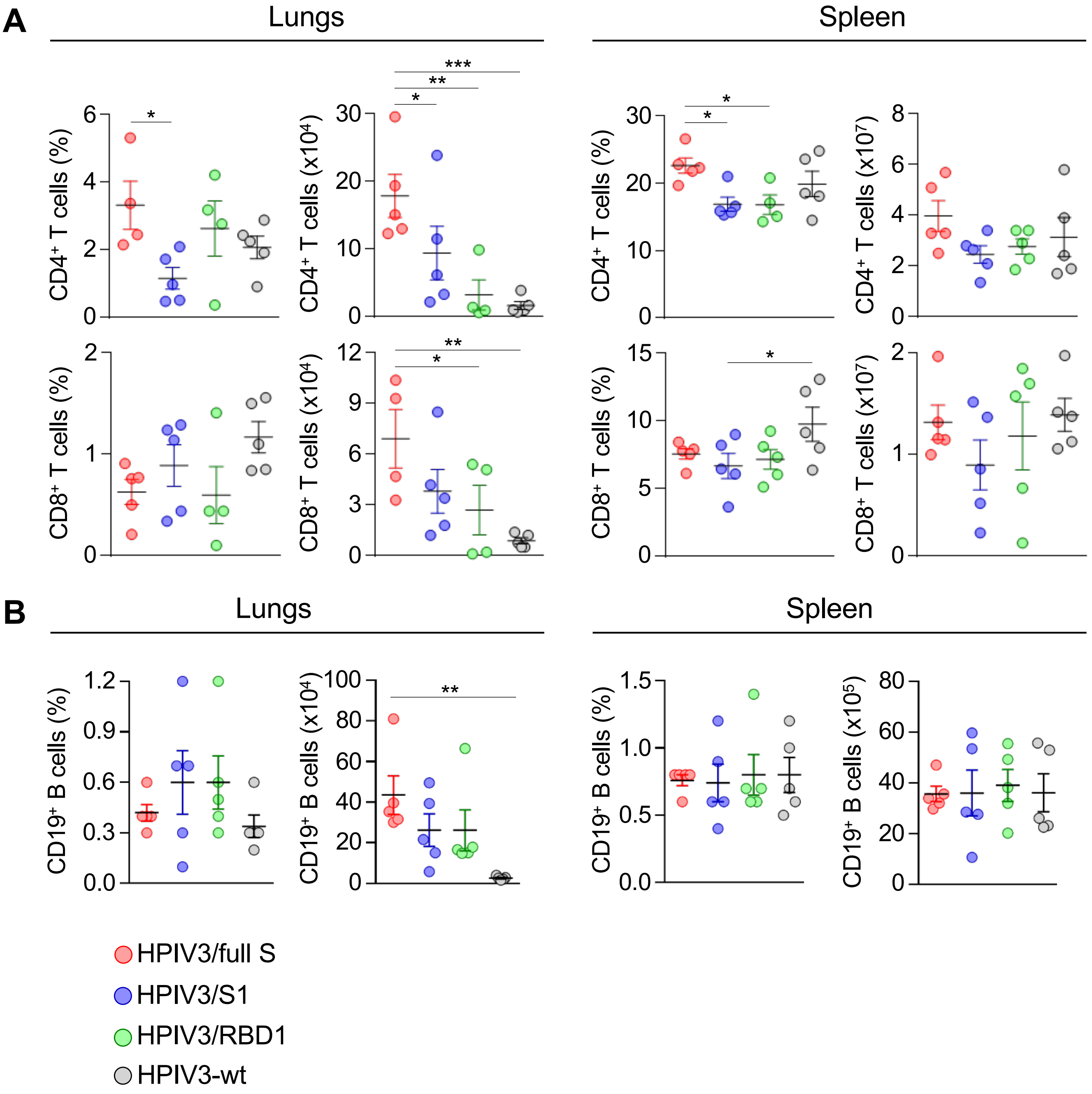

Supplementary Figure 3 (related to Fig. 4)**. Numbers of T and B cells in lungs and spleen at 28 days post vaccination.** Cells were isolated from tissue samples of uninfected animals, stained for T **(A)** or B **(B)** cell markers and analyzed by flow cytometry. The frequency and total numbers of specific cell populations are shown. Data represent mean ± SEM of 4 - 5 animals per group. *p<0.05; **p<0.01; ***p<0.001 (One-way ANOVA with multiple comparisons, Fisher's LSD test).

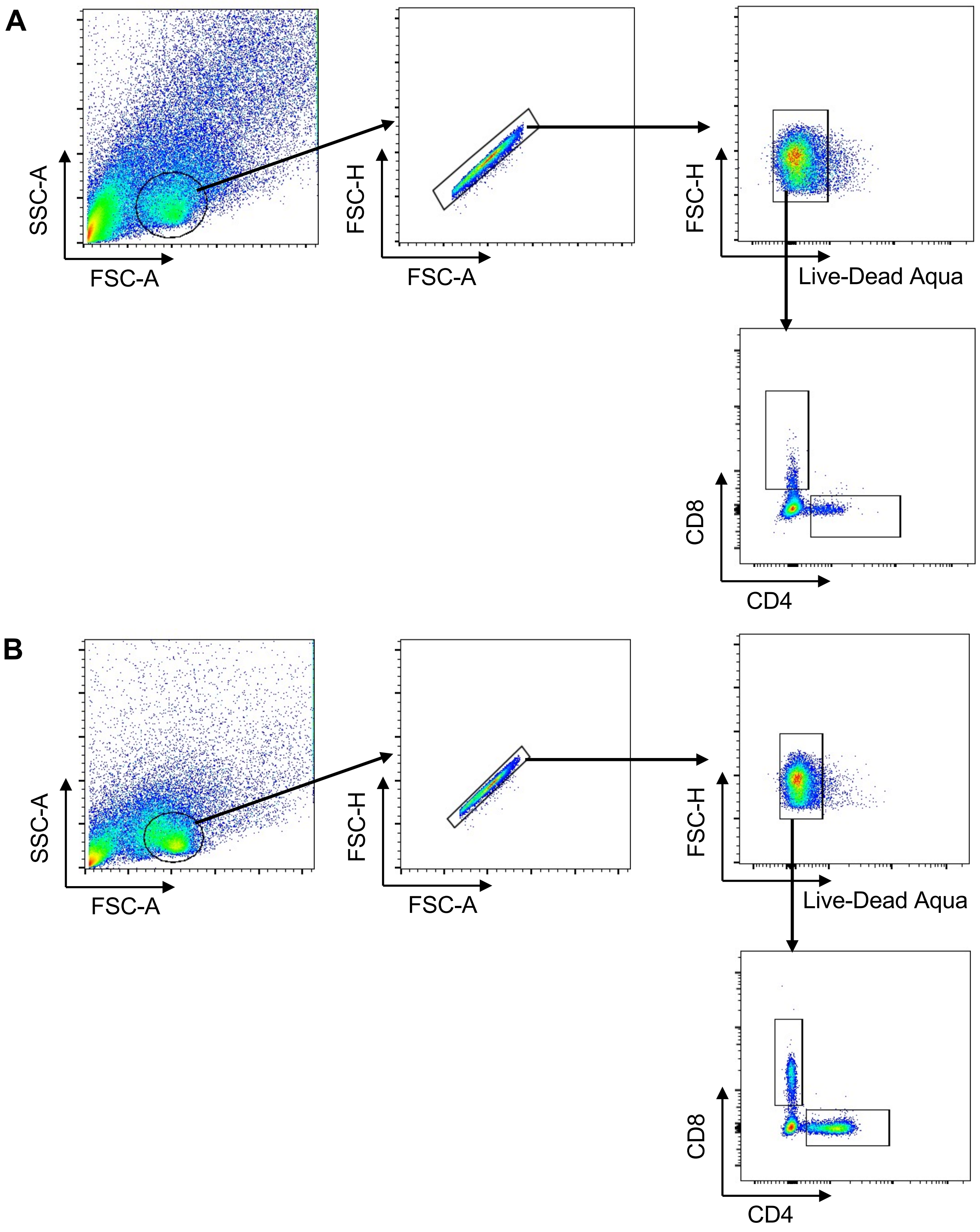

Supplementary Figure 4 (related to Fig. 4). **Flow cytometry gating strategy.** Lung (**A**) and spleen (**B**) cells were isolated from all the groups of hamsters and cultured *in vitro* in the presence of Brefeldin A and GolgiStop with and without S-peptides overnight (see Methods). Cells were collected and stained with Live/Dead Fixable Aqua stain and antibodies specific for T cell markers for 30 min at 4°C in the dark. The cells were fixed in 2% paraformaldehyde, permeabilized and intracellularly stained for IFNγ. The lymphocyte population was gated for singlet and live cells, which were further gated for CD4 and CD8. The $CD4^{+}$ and $CD8^{+}$ cells were further analyzed for IFNγ expression.

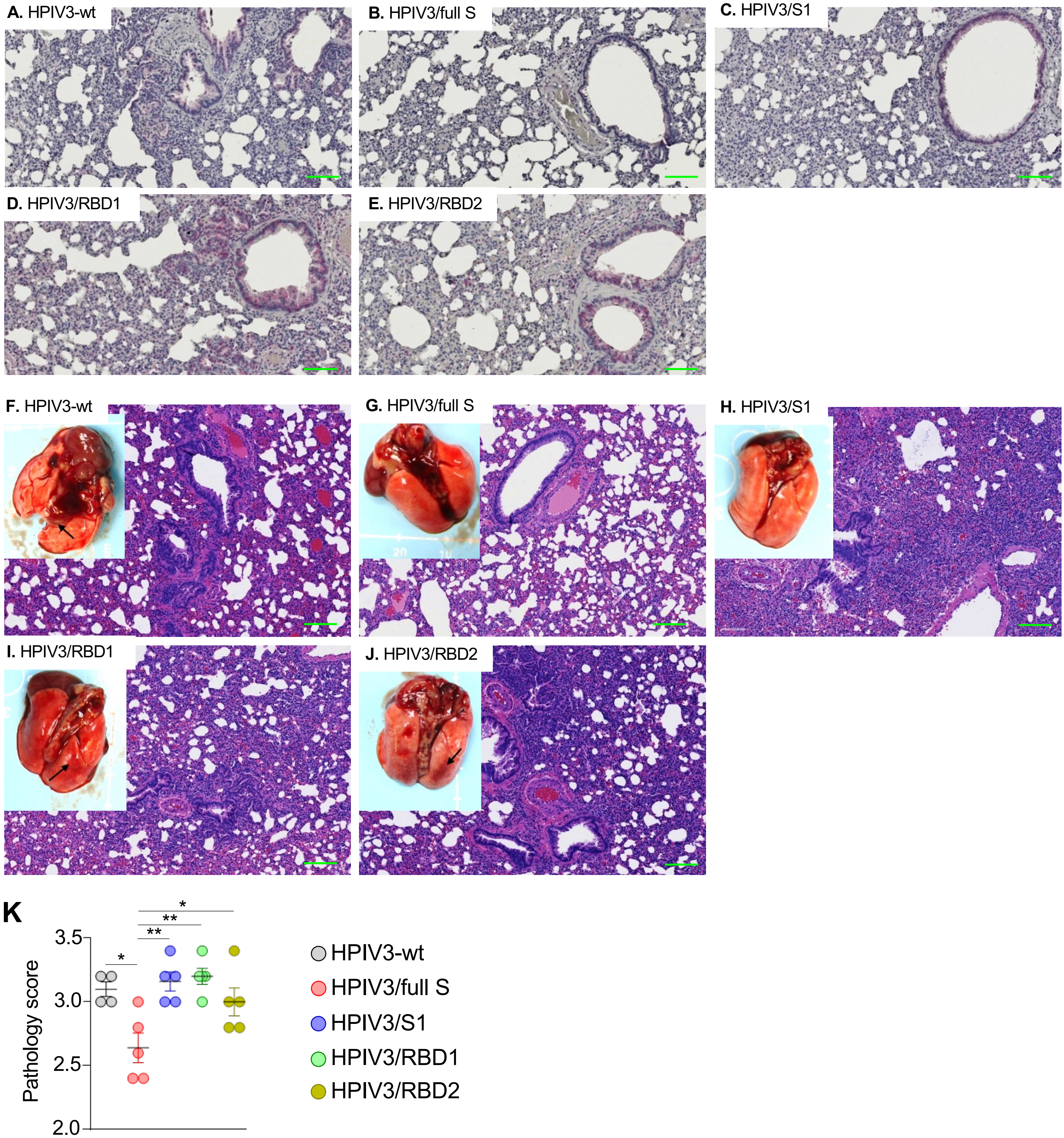

Supplementary Figure 5 (related to Fig. 6)**. Virus-induced lung pathology in vaccinated hamsters on day 14 after infection.** Immunohistochemical staining of SARS-CoV-2 nucleoprotein (NP) antigen in lung tissues (**A-E**) and representative gross and histological lung images (**F-J**). Black arrows show congestion and focal consolidation in the lungs of SARS-CoV-2 infected hamsters. Magnification: 4x. **K**: comparative pathology scores calculated based on criteria described in Table S3. Data represent mean ± SEM of n = 4 – 5 per group. *p<0.01; **p<0.001 (One-way ANOVA with multiple comparisons, Fisher's LSD test). Scale bar: 0.25 µm.

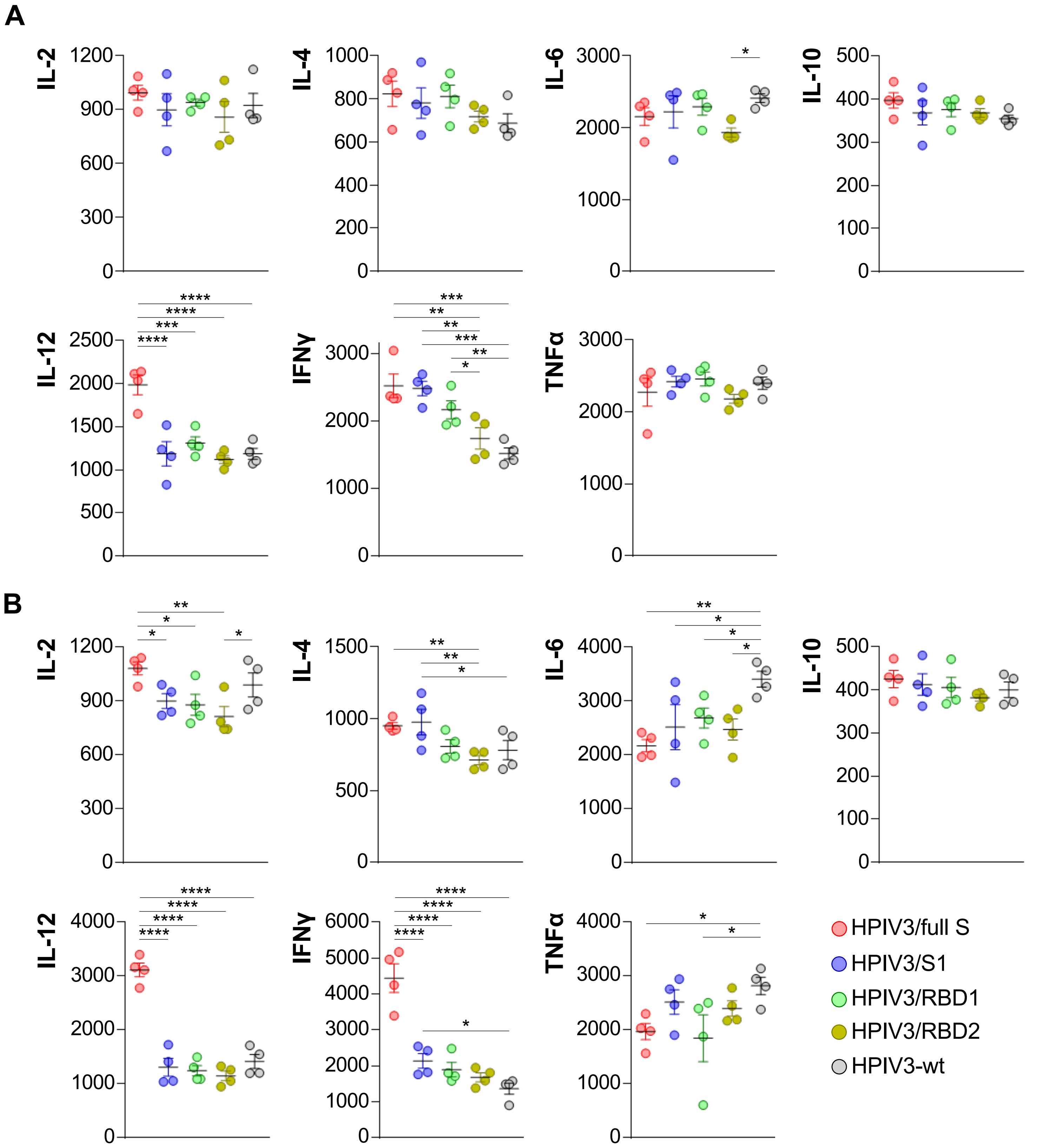

Supplementary Figure 6 (related to Fig. 7)**. Cytokine profile in lungs of vaccinated hamsters following SARS-CoV-2 challenge.** The levels of cytokines (pg/ml) were determined in lung tissue homogenates by ELISA. **A.** Day 3 post-infection. **B.** Day 14 post-infection. Data represent mean ± SEM of 4 animals per group. *p<0.05; **p<0.01; ***p<0.001; ****p<0.0001 (One-way ANOVA with multiple comparisons, Fisher's LSD test).

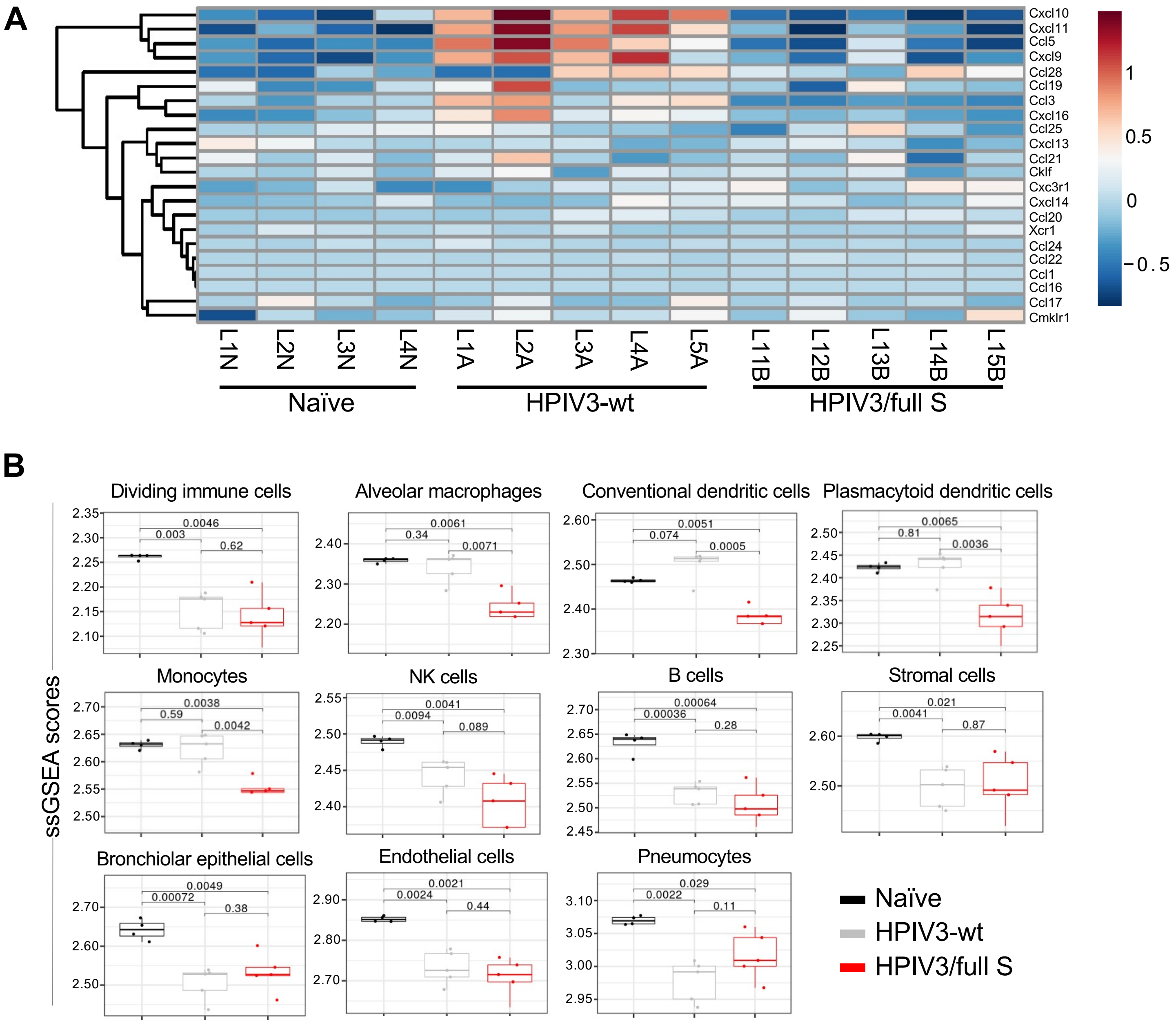

Supplementary Figure 7 (related to Fig. 7)**. HPIV3 vaccine protects hamsters against SARS-CoV-2 infection by downregulating inflammatory response. A.** Heat map of the mRNAs encoding chemokines in hamster lungs from naïve, HPIV3-wt and HPIV3/full S groups. Hierarchical clustering of the genes is shown on the left. The color legend indicates $\log_2$ expression measures. **B.** Cell-type proportions of bulk RNA sequencing data. To evaluate changes in lung cellular composition across the animal groups, the single sample gene-set enrichment analysis (ssGSEA) was applied to quantify the relative abundance of different cell types across samples. ssGSEA scores were tested for differences between sample groups with a two-tailed T-test.

A

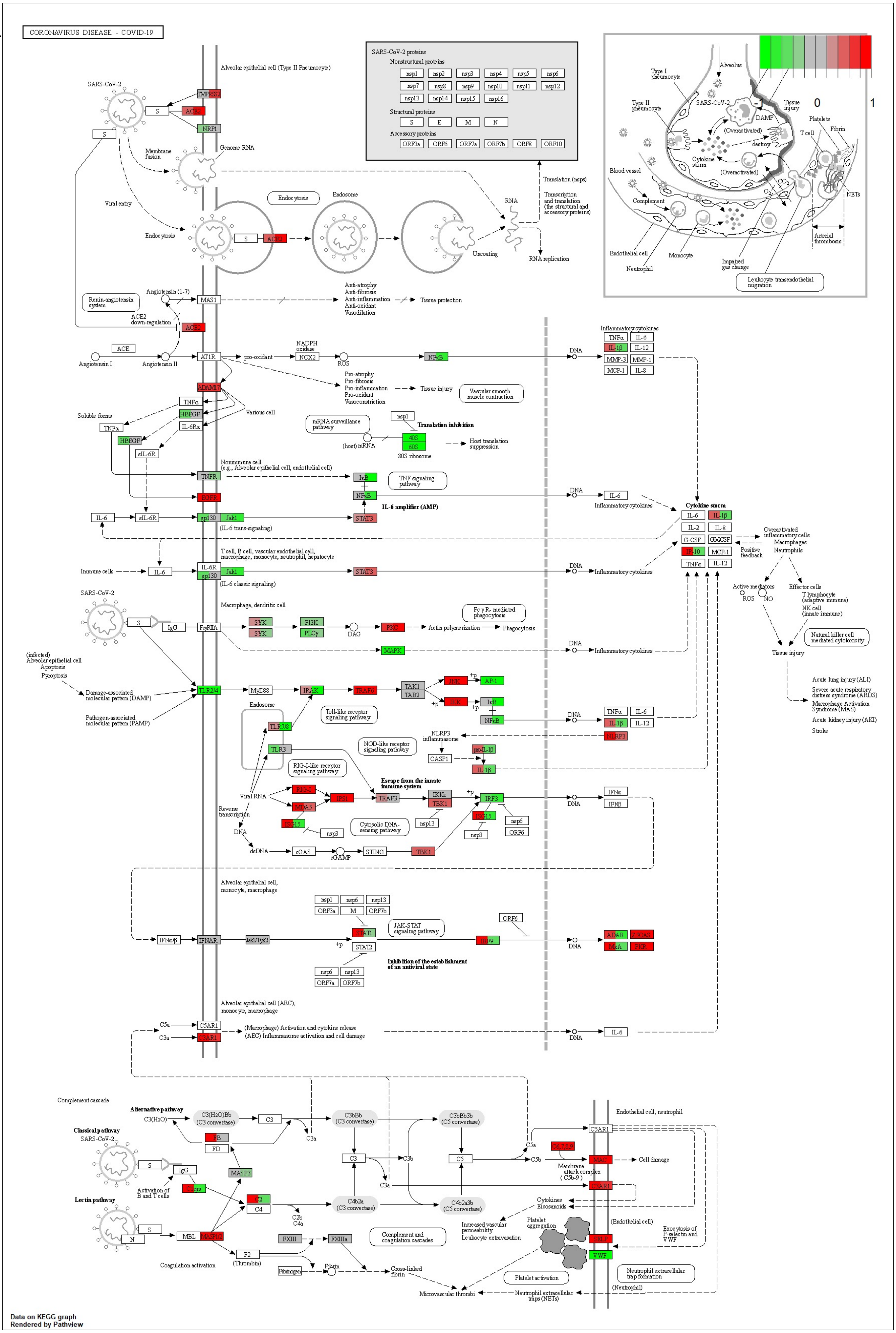
CORONAVIRUS DISEASE - COVID-19
Alveolar epithelial cell (Type II Pneumocyte)
SARS-CoV-2
TMPRSS2
S
ACE2
NRP1
Membrane fusion
Genome RNA
Viral entry
Endocytosis
Endosome
Uncoating
RNA
RNA replication
Translation (nsps)
Transcription and translation (the structural and accessory proteins)
SARS-CoV-2 proteins
Nonstructural proteins
nsp1 nsp2 nsp3 nsp4 nsp5 nsp6
nsp7 nsp8 nsp9 nsp10 nsp11 nsp12
nsp13 nsp14 nsp15 nsp16
Structural proteins
S E M N
Accessory proteins
ORF3a ORF6 ORF7a ORF7b ORF8 ORF10
Alveolus
Type I pneumocyte
Type II pneumocyte
SARS-CoV-2
Tissue injury
DAMP
(Overactivated)
destroy
Platelets
Fibrin
T cell
Cytokine storm
Blood vessel
Complement
NETs
Endothelial cell
Monocyte
Neutrophil
Impaired gas change
Arterial thrombosis
Leukocyte transendothelial migration
0
1
Renin-angiotensin system
Angiotensin (1-7)
MAS1
Anti-atrophy
Anti-fibrosis
Anti-inflammation
Anti-oxidant
Vasodilation
Tissue protection
ACE2 down-regulation
ACE
Angiotensin I
Angiotensin II
AT1R
pro-oxidant
NADPH oxidase
NOX2
ROS
NFκB
DNA
Inflammatory cytokines
TNFα IL-6
IL-1β IL-12
MMP-3 MMP-1
MCP-1 IL-8
Pro-atrophy
Pro-fibrosis
Pro-inflammation
Pro-oxidant
Vasoconstriction
Tissue injury
Vascular smooth muscle contraction
ADAM17
TNFα
HBEGF
IL-6Rα
Various cell
Soluble forms
sIL-6R
mRNA surveillance pathway
nsp1
Translation inhibition
40S
60S
80S ribosome
(host) mRNA
Host translation suppression
Nonimmune cell (e.g., Alveolar epithelial cell, endothelial cell)
TNFR
IκB
TNF signaling pathway
EGFR
IL-6
gp130
Jak1
STAT3
IL-6 amplifier (AMP)
(IL-6 trans-signaling)
Cytokine storm
IL-6 IL-1β
IL-2 IL-8
G-CSF GMCSF
IP-10 MCP-1
TNFα IL-12
Overactivated inflammatory cells
Macrophages
Neutrophils
Positive feedback
Active mediators
ROS NO
Effector cells
T lymphocyte (adaptive immune)
NK cell (innate immune)
Natural killer cell mediated cytotoxicity
Tissue injury
Acute lung injury (ALI)
Severe acute respiratory distress syndrome (ARDS)
Macrophage Activation Syndrome (MAS)
Acute kidney injury (AKI)
Stroke
T cell, B cell, vascular endothelial cell, macrophage, monocyte, neutrophil, hepatocyte
Immune cells
IL-6R
(IL-6 classic signaling)
Macrophage, dendritic cell
IgG
FcγRIIA
SYK
PI3K
PLCγ
DAG
PKC
Actin polymerization
Phagocytosis
Fcγ R- mediated phagocytosis
MAPK
(infected) Alveolar epithelial cell
Apoptosis
Pyroptosis
Damage-associated molecular pattern (DAMP)
Pathogen-associated molecular pattern (PAMP)
TLR2/4
MyD88
IRAK
TRAF6
TAK1
TAB2
JNK
AP-1
IKK
+p
Toll-like receptor signaling pathway
TLR7/8
TLR3
NLRP3 inflammasome
NLRP3
CASP1
pro-IL-1β
IL-1β
NOD-like receptor signaling pathway
RIG-I-like receptor signaling pathway
Escape from the innate immune system
Viral RNA
RIG-I
MDA5
IPS1
TRAF3
IKKε
TBK1
IRF3
ISG15
nsp3
nsp13
nsp6
ORF6
IFNα
IFNβ
Reverse transcription
cGAS
cGAMP
STING
Cytosolic DNA-sensing pathway
dsDNA
Alveolar epithelial cell, monocyte, macrophage
IFNα/β
IFNAR
Jak1/Tyk2
ORF3a M ORF7b
STAT1
STAT2
JAK-STAT signaling pathway
IRF9
ADAR 2'-5'OAS
MxA PKR
Inhibition of the establishment of an antiviral state
ORF7a
Alveolar epithelial cell (AEC), monocyte, macrophage
C5a
C3a
C5AR1
C3AR1
(Macrophage) Activation and cytokine release
(AEC) Inflammasome activation and cell damage
Complement cascade
Alternative pathway
C3(H2O)
C3(H2O)Bb (C3 convertase)
C3
C3bBb (C3 convertase)
C3bBb3b (C5 convertase)
CFB
FD
Classical pathway
MASP3
C1qrs
Activation of B and T cells
Lectin pathway
MBL
MASP1/2
C2
C4
C2b
C4a
C4b2a (C3 convertase)
C4b2a3b (C5 convertase)
C3b
C5
C5b
C6,7,8,9
MAC
Membrane attack complex (C5b-9)
Cell damage
Endothelial cell, neutrophil
Cytokines
Eicosanoids
Increased vascular permeability
Leukocyte extravasation
Platelet aggregation
(Endothelial cell)
SELP
VWF
Exocytosis of P-selectin and VWF
FXIII
FXIIIa
Complement and coagulation cascades
F2
(Thrombin)
Coagulation activation
Fibrinogen
Fibrin
Cross-linked fibrin
Microvascular thrombi
Platelet activation
Neutrophil extracellular trap formation
Neutrophil extracellular traps (NETs)
(Neutrophil)
Data on KEGG graph
Rendered by Pathview

B

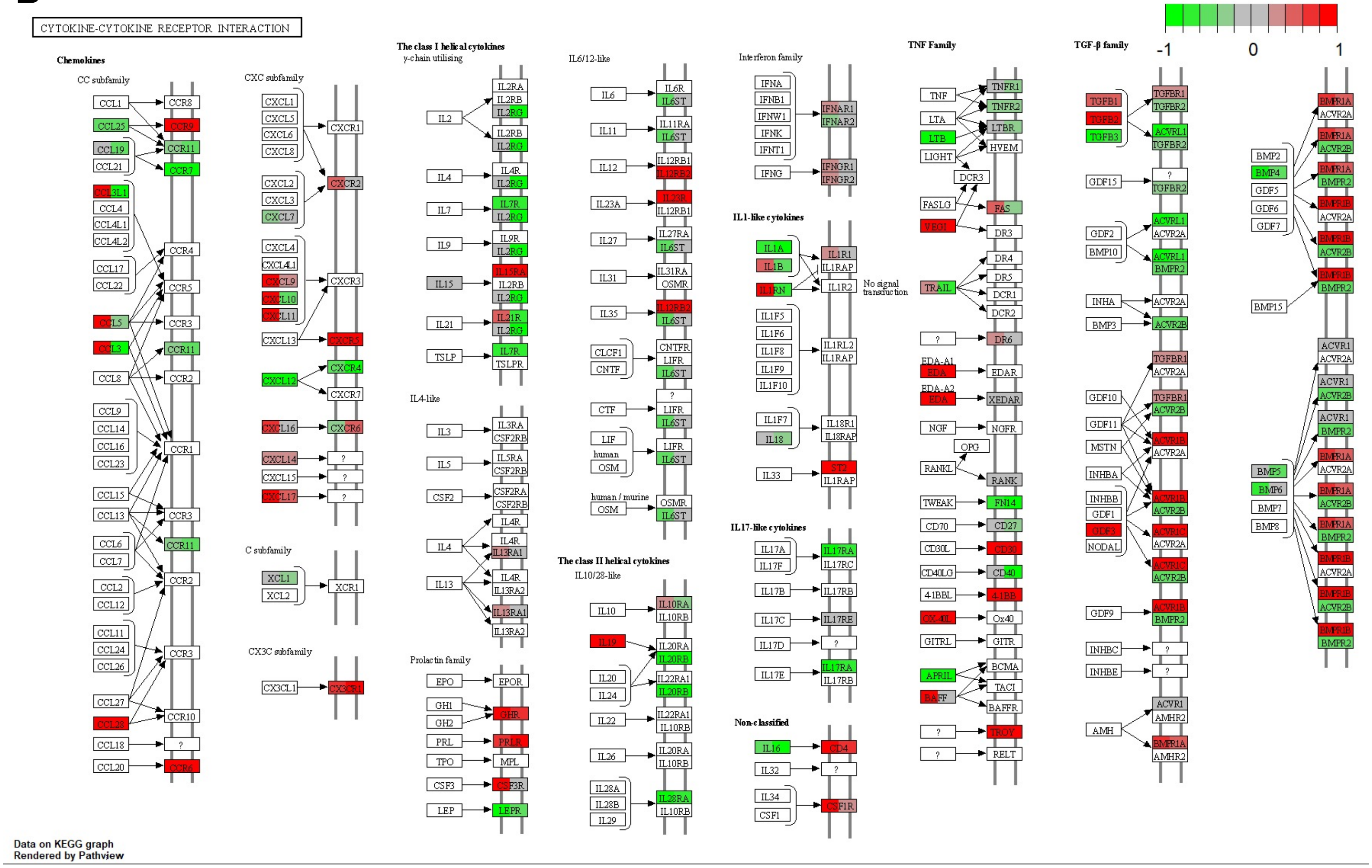

Supplementary Figure 8 (related to Fig. 7)**. The effect of the vaccination on Coronavirus Disease – COVID-19 (A) and Cytokine-Cytokine Receptor Interaction (B) pathways in lungs at 3 dpi.** Each rectangle includes two sections, which show the level of expression of a specific molecule involved in a pathway following inoculation with HPIV3-wt or HPIV3/full S, respectively, as compared to naïve animals.

Supplementary Table 1 (related to Fig. 3). Characteristics of individual pre-challenge serum samples from HPIV3/full S_II group hamsters. The samples are ordered according to their neutralizing titers, from highest to lowest.

Animal ID	Log_{10} Neutralizing titer*	Log_{10} S IgG titer**	Peptides (MFI, relative units)					
			#101	#102	#168	#204	#245	#252
11B	3.383	5.046	***	***	***	7448	***	***
15B	2.897	4.700	***	***	***	11808	***	***
18B	2.794	4.600	***	***	43856	***	***	10276
17B	2.769	4.600	***	***	***	***	***	20213
20B	2.741	4.900	***	***	***	***	***	36723
16B	2.731	4.900	***	***	***	7140	9705	17394
13B	2.650	4.900	***	***	11135	***	***	***
19B	2.506	4.800	6564	8570	***	***	***	7445
12B	2.401	4.600	***	***	***	7498	***	14607
14B	2.173	4.600	***	***	***	***	***	6467

*Fig. 2B data

**Fig. 2A data

***Below the background level

Supplementary Table 2 (related to Fig. 6, Supplementary Fig. 5). Intensity of immunostaining pattern for viral antigen.

Vaccine construct	3 dpi	14 dpi
HPIV3-wt	++/+++	-/+
HPIV3/full S_II	+/++	-/+
HPIV3/S1_II	++/+++	-/+
HPIV3/RBD1_II	++/+++	+/++
HPIV3/RBD2_II	+++	+/++

Notes: - none, + mild (<10% of the positive staining area under 4x field), ++ moderate (10-30% of the positive staining area under 4x field), +++ severe (>30% of the positive staining area under 4x field).

Supplementary Table 3 (related to Fig. 6, Supplementary Fig. 5). Criteria for histopathology scoring.

	**Scores→**	**0**	**1**	**2**	**3**	**4**
**A**	Extent of inflammation (% tissue involved)	0	<10	10-30	30-60	>60
**B**	Inflammatory foci type	No inflammation	Patchy inflammatory foci, few (<2)	Patchy inflammatory foci, many (>2)	Large inflammatory foci, few (<2)	Large inflammatory foci, many (>2)
**C**	Alveolar septa	Thin and delicate	Thickened in <10% HPF	Thickened in <30% HPF	Thickened in <60% HPF	Thickened in >60% HPF
**D**	Airways	Clear; no cells	Few cells in airway	Moderate cells in airway	More cells in air way; Epithelial hyperplasia	Occlusion of air way/epithelial hyperplasia or desquamation
**E**	Alveoli/ perivascular cuff/blood vessels/ pleuritis/cell types	Clear; no inflammatory cells	Few cells. Few PMN or MNC	Moderate cells/ PVC/mild congestion/ mild pleuritis/mostly MNC	More cells/PVC/ more congestion and pleuritis/more MNC and PMN	Abundant cells/large PVC/severe congestion or pleuritis/mixed cells

The criteria were adapted from ref. [1]. HPF – high power field (>10x); PMN – polymorphonuclear cells/heterophils; MNC – mononuclear cells including lymphocytes and macrophages; PVC – perivascular cuff.

REFERENCE

1 Matute-Bello, G. *et al.* An official American Thoracic Society workshop report: features and measurements of experimental acute lung injury in animals. *Am J Respir Cell Mol Biol* **44**, 725-738, doi:10.1165/rcmb.2009-0210ST (2011).
