# Supplementary material for: A single intranasal dose of human parainfluenza virus type 3-vectored vaccine induces effective antibody and memory T cell response in the lungs and protects hamsters against SARS-CoV-2
Source: NPJ Vaccines. 2022 Apr 25;7:47. doi: 10.1038/s41541-022-00471-3 (PMC9038905; doi:10.1038/s41541-022-00471-3)
Supplement: Supplementary file 2 — REPORTING SUMMARY [file 41541_2022_471_MOESM2_ESM.pdf]

nature portfolio

Corresponding author(s): Alexander Buklreyev

Last updated by author(s): Alexander Buklreyev

Reporting Summary

Nature Portfolio wishes to improve the reproducibility of the work that we publish. This form provides structure for consistency and transparency in reporting. For further information on Nature Portfolio policies, see our Editorial Policies and the Editorial Policy Checklist.

Statistics

For all statistical analyses, confirm that the following items are present in the figure legend, table legend, main text, or Methods section.

n/a	Confirmed	
☐	☒	The exact sample size (*n*) for each experimental group/condition, given as a discrete number and unit of measurement
☒	☐	A statement on whether measurements were taken from distinct samples or whether the same sample was measured repeatedly
☐	☒	The statistical test(s) used AND whether they are one- or two-sided<br>*Only common tests should be described solely by name; describe more complex techniques in the Methods section.*
☒	☐	A description of all covariates tested
☒	☐	A description of any assumptions or corrections, such as tests of normality and adjustment for multiple comparisons
☒	☐	A full description of the statistical parameters including central tendency (e.g. means) or other basic estimates (e.g. regression coefficient) AND variation (e.g. standard deviation) or associated estimates of uncertainty (e.g. confidence intervals)
☐	☒	For null hypothesis testing, the test statistic (e.g. *F*, *t*, *r*) with confidence intervals, effect sizes, degrees of freedom and *P* value noted<br>*Give P values as exact values whenever suitable.*
☒	☐	For Bayesian analysis, information on the choice of priors and Markov chain Monte Carlo settings
☒	☐	For hierarchical and complex designs, identification of the appropriate level for tests and full reporting of outcomes
☒	☐	Estimates of effect sizes (e.g. Cohen's *d*, Pearson's *r*), indicating how they were calculated

Our web collection on statistics for biologists contains articles on many of the points above.

Software and code

Policy information about availability of computer code

Data collection	n/a
Data analysis	n/a

For manuscripts utilizing custom algorithms or software that are central to the research but not yet described in published literature, software must be made available to editors and reviewers. We strongly encourage code deposition in a community repository (e.g. GitHub). See the Nature Portfolio guidelines for submitting code & software for further information.

Data

Policy information about availability of data

All manuscripts must include a data availability statement. This statement should provide the following information, where applicable:

- Accession codes, unique identifiers, or web links for publicly available datasets
- A description of any restrictions on data availability
- For clinical datasets or third party data, please ensure that the statement adheres to our policy

The datasets generated during the current study are available from the corresponding author on reasonable request. RNA sequencing data have been deposited in NCBI’s Gene Expression Omnibus and are accessible through the GEO Series accession number GSE193288.

Field-specific reporting

Please select the one below that is the best fit for your research. If you are not sure, read the appropriate sections before making your selection.

☒ Life sciences ☐ Behavioural & social sciences ☐ Ecological, evolutionary & environmental sciences

For a reference copy of the document with all sections, see nature.com/documents/nr-reporting-summary-flat.pdf

Life sciences study design

All studies must disclose on these points even when the disclosure is negative.

Sample size	3 – 10
Data exclusions	n/a
Replication	n/a
Randomization	n/a
Blinding	n/a

Behavioural & social sciences study design

All studies must disclose on these points even when the disclosure is negative.

Study description	n/a
Research sample	n/a
Sampling strategy	n/a
Data collection	n/a
Timing	n/a
Data exclusions	n/a
Non-participation	n/a
Randomization	n/a

Ecological, evolutionary & environmental sciences study design

All studies must disclose on these points even when the disclosure is negative.

Study description	n/a
Research sample	n/a
Sampling strategy	n/a
Data collection	n/a
Timing and spatial scale	n/a
Data exclusions	n/a
Reproducibility	n/a
Randomization	n/a
Blinding	n/a

Did the study involve field work? ☐ Yes ☒ No

Reporting for specific materials, systems and methods

We require information from authors about some types of materials, experimental systems and methods used in many studies. Here, indicate whether each material, system or method listed is relevant to your study. If you are not sure if a list item applies to your research, read the appropriate section before selecting a response.

Materials & experimental systems

n/a	Involved in the study
☐	☒ Antibodies
☐	☒ Eukaryotic cell lines
☒	☐ Palaeontology and archaeology
☐	☒ Animals and other organisms
☒	☐ Human research participants
☒	☐ Clinical data
☒	☐ Dual use research of concern

Methods

n/a	Involved in the study
☒	☐ ChIP-seq
☐	☒ Flow cytometry
☒	☐ MRI-based neuroimaging

Antibodies

Antibodies used

1) Rabbit anti-HPIV3 polyclonal (Bioqual MS-456; provided by Drs. Peter Collins and Ursula Buchholz, NIAID)
2) Goat anti-Rabbit IgG (H+L) Secondary Antibody, HRP (ThermoFisher Scientific, Cat. #65-6120)
3) Goat Anti-Syrian Hamster IgG H&L (HRP) preadsorbed (Abcam, Cat. #ab7146)
4) Goat Anti-Syrian Hamster IgG H&L (Cy5 ®) preadsorbed (Abcam, Cat. #ab6568, Lot #GR202007-5)
5) Actin Monoclonal Antibody (ACTN05 (C4)) (Invitrogen, Cat. #MA5-11869)
6) SARS-CoV-2 (2019-nCoV) Spike Antibody, Rabbit PAb, Antigen Affinity Purified (Sino Biological, Cat. #40589-T62)
7) IRDye® 680RD Goat anti-Mouse IgG Secondary Antibody (LI-COR, Cat. #926-68070, Lot #C60405_08)
8) IRDye® 800CW Goat anti-Rabbit IgG Secondary Antibody (LI-COR, Cat. #925-32211, Lot #C61103-03)
9) SARS-CoV-2 (COVID-19) nucleocapsid antibody (GeneTex, Cat. #GTX135357)
10) ImmPRESS®-AP Horse Anti-Rabbit IgG AP (Vector Laboratories, Cat. #MP-5401-50)
11) Mouse CD4-FITC (BioLegend, clone GK1.5, Cat. #100406, Lot #B284855)
12) Rat CD8-PE (Invitrogen, clone eBio341, Cat. #12-0080-82, Lot #2254786)
13) Mouse CD44-Pacific Blue (BioLegend, clone IM7, Cat. #103020, Lot #B293621)
14) Mouse CD62L-APC/Fire750 (BioLegend, clone MEL-14, Cat. #104450, Lot #B287189)
15) Mouse CD25 PE/cy7 (BioLegend, clone PC61, Cat. #102010, Lot #B320333)
16) Mouse IFNγ-APC (BioLegend, clone XMG1.2, Cat. #505810, Lot #B290393)
17) Mouse B220-BV421 (BioLegend, clone RA3-6B2, Cat. #103240, Lot #B316684)
18) Mouse CD19-PerCP (BioLegend, clone 4G7, Cat. #392510, Lot #B317125)

Validation

Validation of IFNγ-specific antibody (BioLegend, clone XMG1.2) for intracellular cytokine staining (ICS; also validated in PMID27552546):
Spleen cells were isolated from naïve hamsters and cultured in vitro. The cells were cultured in presence of GolgiStop/Brefeldin A with and without PMA/Ionomycin (5 ng/ml/200ng/ml) for overnight. Cells were collected and surface stained for CD4 and CD8 markers. After fixation, cells were permeabilized and ICS was done for IFNγ cytokine. Compared to unstimulated cells, the PMA/Ionomycin stimulated cells had significantly higher numbers of CD4+IFNγ+ cells. As well, when PMA/Ionomycin-stimulated cells stained with anti-IFNγ antibody, IFNγ-specific cells are clearly observed, while control without IFNγ antibody shows no specific-stained cells.

Eukaryotic cell lines

Policy information about cell lines

Cell line source(s)

Vero-E6 (ATCC: CRL-1586)
LLC-MK2 (ATCC: CCL-7)

Authentication

None of the cell lines used were authenticated by any method other than microscopic observation

Mycoplasma contamination

All cell lines tested negative for mycoplasma contamination

Commonly misidentified lines (See ICLAC register)

n/a

Animals and other organisms

Policy information about studies involving animals; ARRIVE guidelines recommended for reporting animal research

Laboratory animals

Species: Hamsters
Strain: Syrian Golden

	Sex: Female<br>Age: 5-6 weeks<br>Vendor: Envigo
Wild animals	n/a
Field-collected samples	n/a
Ethics oversight	The animal protocol for testing of HPIV3-based vaccine constructs against SARS-CoV-2 in hamsters was approved by the Institutional Animal Care and Use Committee (IACUC) of the University of Texas Medical Branch in compliance with the Animal Welfare Act and other applicable federal statutes and regulations relating to animals and experiments involving animals.

Note that full information on the approval of the study protocol must also be provided in the manuscript.

Flow Cytometry

Plots

Confirm that:

☒ The axis labels state the marker and fluorochrome used (e.g. CD4-FITC).

☒ The axis scales are clearly visible. Include numbers along axes only for bottom left plot of group (a 'group' is an analysis of identical markers).

☒ All plots are contour plots with outliers or pseudocolor plots.

☒ A numerical value for number of cells or percentage (with statistics) is provided.

Methodology

Sample preparation	Single cell suspensions were prepared from the lungs and spleens of vaccinated and control hamsters. Briefly, the lungs were cut into small pieces and suspended in 1 ml digestion buffer containing 3 mg/ml collagenase type II and 40 U/ml rDNase I (Worthington Biochemical). Following digestion for 40 min at 37°C, the lung cells were passed through 70 µm cell strainer and collected as pellet by centrifugation (250 x g, 10 min). Red blood cells were lysed with ACK lysis buffer (Lonza) and washed out with an excess of 0.5% BSA in 1x PBS (PBS/BSA). Cells were further processed for gradient centrifugation using Histopaque-1077 (Sigma-Aldrich) to enrich immune cells, following standard procedure. The final cell pellet was resuspended in PBS/BSA and cells were counted in TC20TM Automated Cell Counter (Bio-Rad Laboratories). The spleen samples were gently minced with syringe plunger on top of the cell strainer and red blood cells were lysed as above. The cells were resuspended in PBS/BSA buffer and counted.
Instrument	LSR Fortessa (BD)
Software	FlowJo v10.8
Cell population abundance	n/a
Gating strategy	Live lymphocyte populations were gated for CD4 and CD8, which were futher gated for IFNg. As well, the lymphocyte populations were gated for CD19 and B220 for identification of B cells.

☐ Tick this box to confirm that a figure exemplifying the gating strategy is provided in the Supplementary Information.
